# From pathogenic to reparative: Context-Dependent function of extracellular vesicles in osteoarthritis

**DOI:** 10.1016/j.bioactmat.2026.09.005

**Published:** 2026-09-08

**Authors:** Zhen Yang, Qiyuan Lin, Hao Li, Senyang Xiao, Hebin Ma, Jianhao Lin, Dan Xing

**Affiliations:** a Arthritis Clinical and Research Center, Peking University People's Hospital, No.11 Xizhimen South Street, Beijing, 100044, China; b Arthritis Institute, Peking University, Beijing, 100044, China; c School of Medicine, Nankai University, Tianjin, China; d Department of Traumatology, Orthopedics and Disaster Surgery, I.M. Sechenov First Moscow State Medical University (Sechenov University), Russia

**Keywords:** Extracellular vesicles, Osteoarthritis, Intercellular communication, Mesenchymal stromal cells, Biomarkers

## Abstract

Osteoarthritis (OA) is a whole-joint disease characterized by progressive structural degeneration and chronic low-grade inflammation affecting the cartilage, synovium, subchondral bone, and immune compartments. It is a complex degenerative disorder associated with substantial morbidity, heterogeneous clinical trajectories, and limited disease-modifying treatment options. Extracellular vesicles (EVs) have emerged as important mediators of intercellular communication within the OA joint microenvironment, and are implicated in pathophysiological responses to mechanical stress, inflammatory cues, and metabolic dysfunction. Through the transfer of context-dependent nucleic acid, protein, and lipid cargoes, EVs can amplify pathogenic processes in OA such as synovitis, cartilage catabolism, and cellular senescence, while also supporting reparative pathways. Understanding the mechanisms governing EV biogenesis, cargo selection, tissue targeting, and functional heterogeneity offers opportunities to identify mechanistically informed biomarkers and therapeutic strategies. This review discusses emerging concepts in EV-mediated joint communication, highlights translational potential and limitations, and outlines key priorities for advancing EV-based diagnostics and therapies in OA. However, EV-based strategies remain largely at the preclinical or experimental stage, and rigorous validation is required before they can enter routine clinical practice.

## Highlights

• OA is a whole-joint disease in which cartilage degeneration, synovial inflammation, subchondral bone remodeling and immune activation are tightly interconnected rather than isolated pathological events.
• EVs act as central mediators of intercellular communication within the osteoarthritic joint by transferring context-dependent RNA, protein and lipid cargoes between resident and infiltrating cell populations.
• EV-mediated signaling is highly heterogeneous and dynamically regulated by cellular origin, joint microenvironment and disease stage, giving rise to both pathogenic EV programmes that amplify inflammation and matrix degradation and reparative EV programmes that support tissue homeostasis.
• Advances in single-vesicle analysis and multi-omics profiling have enabled the identification of functionally distinct EV subtypes and have revealed coordinated EV-driven communication networks linking chondrocytes, synoviocytes, immune cells and subchondral bone–associated cells.
• Dissecting the mechanisms governing EV biogenesis, cargo selection, tissue targeting and functional outcomes is essential for understanding OA pathophysiology and for the rational development of EV-informed biomarkers and therapeutic strategies.

## Graphical abstract

This review examines extracellular vesicle (EV)–mediated communication across the osteoarthritic joint, highlighting diverse cellular sources and cargo mechanisms. It discusses how environmental context directs EVs toward either a pathogenic program driving degeneration or a reparative program facilitating tissue homeostasis and repair.

**Figure:**
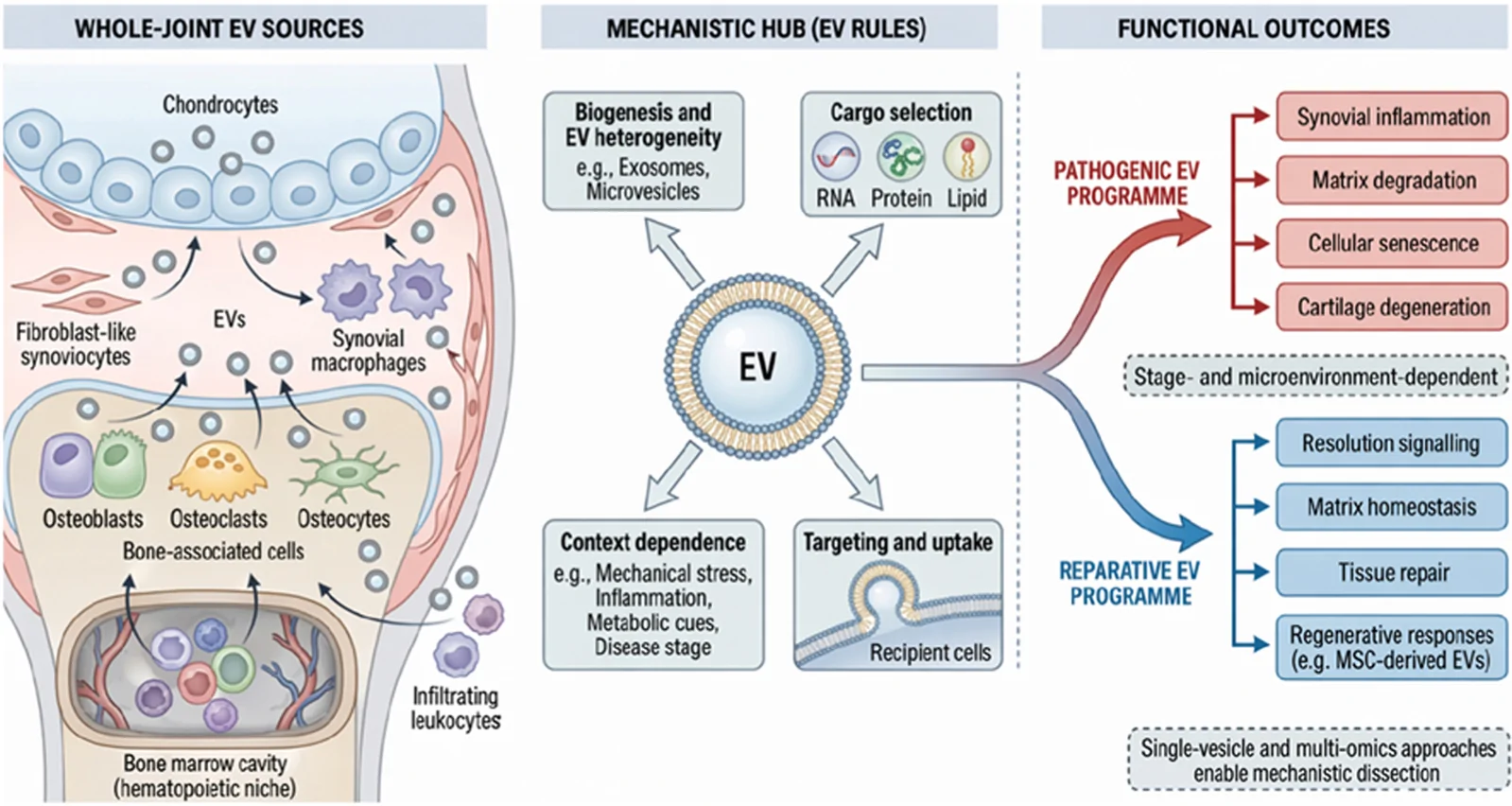

**Key points:**

● OA is a whole-joint disease in which cartilage degeneration, synovial inflammation, subchondral bone remodeling, and immune activation are tightly interconnected rather than isolated pathological events.
● EVs act as key mediators of intercellular communication within the osteoarthritic joint by transferring context-dependent RNA, protein, and lipid cargoes between resident and infiltrating cell populations.
● EV-mediated signaling is highly heterogeneous and dynamically regulated by cellular origin, joint microenvironment, and disease stage, giving rise to both pathogenic EVs that amplify inflammation and matrix degradation and reparative EVs that support tissue homeostasis.
● Advances in single-vesicle analysis and multi-omics profiling have enabled the identification of functionally distinct EV subtypes and have revealed coordinated EV-driven communication networks linking chondrocytes, synoviocytes, immune cells, and subchondral bone–associated cells.
● Dissecting the mechanisms governing EV biogenesis, cargo selection, tissue targeting, and functional outcomes is essential for understanding OA pathophysiology and for the rational development of EV-informed biomarkers and therapeutic strategies.

## 1 Introduction

Osteoarthritis (OA) has undergone a profound conceptual evolution from a simplistic “wear-and-tear” cartilage disorder to a dynamic, multicellular disease of the entire joint. Contemporary understanding positions OA as a complex pathological network involving articular cartilage degradation, synovial inflammation, subchondral bone remodeling, and alterations in periarticular tissues such as the infrapatellar fat pad and meniscus [1,2]. This holistic perspective underscores that disease progression arises not from isolated tissue failure but from dysregulated crosstalk among chondrocytes, osteoblasts, synoviocytes, macrophages, mesenchymal stromal cells (MSCs), and immune cells within the joint microenvironment [3,4]. The functional integrity of the joint thus depends on precisely coordinated intercellular dialogues, which are increasingly recognized as central to both homeostasis and pathology.

Historically, intercellular communication in OA has been framed predominantly through soluble mediators, including cytokines (e.g., IL-1β, TNF-α), chemokines, growth factors, and matrix-degrading enzymes diffusing through the synovial fluid or extracellular matrix (ECM) [1]. While this framework elucidated key inflammatory and catabolic pathways, it presents critical conceptual limitations. Soluble factors alone cannot fully account for the spatial precision, signal amplification, or multi-molecular cargo delivery observed in OA pathophysiology. The diffusion-dependent kinetics of soluble factors mean they lack the targeting specificity required for selective cellular reprogramming, and they offer limited capacity for the coordinated transfer of functionally integrated biomolecular complexes such as non-coding RNAs, proteins, and lipids [5,6]. Moreover, the transient nature of soluble signals is insufficient to sustain the persistent, progressive tissue remodeling characteristic of OA, suggesting missing mechanistic layers in traditional models.

A network-based view of OA pathogenesis has begun to replace traditional tissue-centric models, emphasizing dynamic communication among cartilage, synovium, subchondral bone, and immune compartments [7]. Within this complex signaling landscape, EVs operate alongside soluble mediators, ECM-derived signals, and direct cell–cell contacts to form a responsive and adaptive intercellular communication system [6,8]. Rather than acting in isolation, EVs integrate multiple contextual cues and propagate coordinated responses across joint tissues [9,10]. Originally dismissed as inert cellular debris, EVs are now recognized as sophisticated, membrane-bound nanocarriers secreted by virtually all joint-resident cells, including chondrocytes, synoviocytes, osteocytes, MSCs, and immune cells [6,10,11]. EVs encapsulate key bioactive cargo (e.g., miRNAs, mRNAs, proteins, lipids) responsible for intercellular communication, protecting them from enzymatic degradation and enabling stable transfer across physiological barriers [5,12]. In a joint microenvironment, EVs mediate both short-range communication within the synovial interstitium and long-distance signaling through the systemic circulation, facilitating crosstalk among articular cartilage, subchondral bone, other joint tissues such as the synovium and immune cells, and distant tissues [13,14]. For instance, osteocyte-derived EVs carrying miR-23b-3p have been shown to modulate chondrocyte metabolism [15], while EVs from fibroblast-like synoviocytes (FLSs) can propagate inflammatory signals to cartilage [6,8], and neutrophil EVs contribute to inflammation resolution. These findings illustrate the key roles of EVs not as isolated messengers within the joint but as dynamic nodes of an integrated communication architecture, and key mediators in OA pathophysiology.

Importantly, EV-mediated signaling is highly plastic [5,16]. OA-related pathological conditions such as ageing-associated cellular senescence or chronic inflammatory priming in obesity can reprogram EV biogenesis and cargo composition, shifting EV function from maintaining tissue homeostasis towards amplifying degeneration [1,3,17]. Senescence-associated secretory phenotypes (SASPs), inflammatory cytokine milieus and altered metabolic states reshape EV cargo profiles, converting regenerative or protective signals into drivers of cartilage breakdown, synovitis, and aberrant bone remodeling. Conversely, EVs derived from young MSCs or experimentally modified stem cell sources have demonstrated the capacity to restore aspects of joint homeostasis by modulating inflammatory pathways, promoting autophagy, and directing immune cells towards reparative phenotypes [18,19]. This functional duality underscores that EVs are not intrinsically pathogenic or therapeutic, but act as context-dependent regulators within the cellular communication network of articulating joints [1,11].

In this review, we synthesize current evidence on EV-mediated intercellular communication in OA from a mechanistic and systems-level perspective, moving beyond reductionist narratives that frame EVs primarily as therapeutic delivery vehicles. We examine how EV signaling intersects with established molecular pathways across joint tissues, dissect cargo-dependent mechanisms influencing chondrocyte senescence, synovial inflammation, and subchondral bone remodeling, and discuss emerging strategies aimed at enhancing EV specificity and functional efficacy. We also highlight key conceptual and technical challenges, including the standardization of EV isolation from complex joint biofluids, the functional dissection of EV subtypes and heterogeneity, and the translation of preclinical insights into clinically meaningful biomarkers and interventions. By situating EVs within the broader framework of whole-joint biology, this review aims to advance a paradigm shift in OA research: from viewing OA as a collection of tissue-specific failures to understanding it as a disorder of dysregulated intercellular communication. While some reviews have surveyed the role of EVs in OA from the perspective of MSC-based therapeutics or individual cargo classes such as miRNAs and proteins [[20], [21], [22]], this review takes a different approach of adopting a unified conceptual framework centered on the context-dependent nature of EV signaling. Specifically, we emphasize that EV function is not an intrinsic property of the cellular source but is dynamically programmed by the donor cell state, disease stage, and local joint microenvironment. This perspective enables an integrated analysis of how the same EV populations can transition between pathogenic and reparative functions across OA progression, and how this plasticity can be rationally exploited for biomarker development and therapeutic engineering. Throughout, we anchor this framework in recent technological advances including single-vesicle analysis and multi-omics profiling that have begun to illuminate EV heterogeneity at unprecedented resolution.

## 2 Fundamental properties and context-dependent behavior of EVs

### 2.1 EV classification, biogenesis and core properties

EVs are lipid bilayer-enclosed particles released by virtually all cell types into biofluids including synovial fluid, plasma, and conditioned culture medium [[23], [24], [25], [26]]. They are conventionally categorized by biogenetic origin into three principal subtypes: exosomes (∼50–150 nm), produced through the endosomal system by inward budding of multivesicular bodies (MVBs) to form intraluminal vesicles (ILVs), followed by MVB–plasma membrane fusion and release [27,28]; microvesicles (also termed ectosomes; 100–1000 nm), generated by direct outward budding and fission of the plasma membrane [29]; and apoptotic bodies (>1000 nm), released as large membranous fragments during programmed cell death [30]. The recent discovery of additional EV subtypes including migrasomes has further underscored a remarkable heterogeneity of EV populations [30,31]. Given that routine isolation methods cannot definitively resolve EV biogenetic origin, MISEV recommendations from the International Society of Extracellular Vesicles (ISEV) suggest size-based operational definitions, namely small EVs (sEVs, <200 nm) and medium/large EVs (>200 nm), to minimize ambiguity arising from inconsistent nomenclature [32,33].

EV biogenesis proceeds through multiple mechanistically distinct routes that directly determine cargo composition and functional properties. Exosome formation is driven by both endosomal sorting complex required for transport (ESCRT)-dependent and ESCRT-independent mechanisms: the former involves the sequential action of ESCRT-0/I/II/III complexes, the accessory protein ALIX, and the AAA ATPase VPS4 [34,35]; the latter encompasses the syndecan–syntenin–ALIX pathway [36], tetraspanin-enriched microdomains (CD63, CD81, CD9) [37], and ceramide-dependent pathways mediated by neutral sphingomyelinase 2 (nSMase2) [38]. Microvesicle shedding is regulated by calcium influx, cytoskeletal reorganization [39], and acid sphingomyelinase activity [38], whereas apoptotic body formation involves the caspase 3–ROCK1 signaling axis and actomyosin contractility [40]. These pathways can operate concurrently within the same cell, producing biochemically distinct EV subpopulations with divergent molecular composition and functional properties.

Critically, EV cargo is not a random sample of cytoplasmic contents but is loaded through highly selective sorting mechanisms: RNA-binding proteins (e.g., hnRNPA2B1, YBX1, SYNCRIP, FMRP) mediate the selective incorporation of specific miRNAs and lncRNAs into nascent vesicles [41,42]; post-translational modifications (ubiquitination, SUMOylation, phosphorylation, O-GlcNAcylation) serve as molecular switches governing protein cargo incorporation [43,44]; and lipid microdomain organization influences the partitioning of membrane proteins and specific lipids onto budding vesicles [45]. This selective loading machinery ensures that the molecular composition of EVs directly reflects the immediate state of the parent cell, meaning that the same cell, under different conditions, can produce EVs with markedly different cargo profiles and functional properties. Furthermore, EV isolation and characterization methods fundamentally influence the purity and composition of the resulting EV populations. Hence, methodological divergence can shape experimental outcomes and lead to different interpretations of EV biological function. The MISEV2018 and more recent MISEV2023 guidelines provide the current field-consensus framework for experimental standardization and reporting transparency in EV research.

### 2.2 EV targeting specificity and single-vesicle analysis

The functional specificity of EV-mediated communication depends not only on selective cargo loading but also on the capacity of EVs to recognize and engage defined recipient cells. EV surface proteins are the key molecular drivers of targeting tropism, particularly integrins, tetraspanins, and proteoglycans. Specific integrin heterodimer repertoires confer selectivity for distinct cell types and tissues [46]. In other tissue systems such as the pre-metastatic niche, cancer-associated fibroblast-derived EVs are preferentially taken up by lung fibroblasts through surface α2β1 integrin, while blockade of α2β1 suppresses this targeting and impairs niche formation [47]. Mechanistically, αvβ1 integrin regulates EV binding to fibronectin, thereby modulating EV retention within the ECM and subsequent fibroblast uptake and activation [48]. In the hematopoietic system, mouse hematopoietic stem and progenitor cell-derived EVs target endothelial cells through α4β7 integrin, cooperating with CXCL12-CXCR4 signaling to promote bone marrow homing [49]. Notably, single-vesicle analysis has demonstrated that correct α–β subunit pairing is a prerequisite for organotropic targeting, whereby EVs bearing unpaired integrins or αv-dominated dimers exhibit negligible targeting capacity [46]. These findings have underscored the critical requirement of proper heterodimeric assembly for EV tropism.

In the OA joint, EV targeting specificity carries particular significance. The dense cartilage ECM is enriched in negatively charged aggrecan, presenting a formidable diffusion barrier [50]. Whether intrinsic or delivered EVs can penetrate cartilage to reach resident chondrocytes is governed by their surface charge, size, and surface protein composition. To date, direct experimental evidence identifying specific integrins that mediate EV targeting to chondrocytes in the OA joint is lacking, representing a significant knowledge gap in the field.

Alongside advances in elucidating EV targeting mechanisms, single-vesicle analysis technologies are rapidly progressing to overcome a key limitation of traditional bulk assays, which average signals across populations and mask the functional diversity of individual vesicles. Among these emerging tools, microfluidic technology represents a promising strategy for extracellular vesicle analysis, enabling precise and sensitive detection with minimal sample input and addressing major drawbacks of conventional analytical methods [51]. Other mainstream single-vesicle analytical platforms include: single-particle interferometric reflectance imaging sensing (SP-IRIS), which enables EV sizing, concentration counting, and co-localization analysis of surface markers [52]; nanoflow cytometry, a high-throughput platform capable of simultaneous multi-parameter detection on individual circulating EVs, potentially discriminating disease states from healthy controls [53]; single-vesicle RNA sequencing, leveraging droplet-based platforms such as 10x Genomics to achieve transcriptomic profiling at single-EV resolution, revealing heterogeneity masked in bulk analyses [54]; and proximity barcoding assays (PBA), enabling multiplexed surface protein analysis of individual EVs [55].

These technologies have enabled innovative insights in OA research. For instance, through single-vesicle analysis, a distinctive “hybrid EV” (hEV) subpopulation has been identified in OA synovial fluid, co-expressing both pro-inflammatory macrophage and chondrocyte surface markers [56]. Interestingly, hEV abundance significantly correlates with OA clinical severity, and they induce chondrocyte mitochondrial damage upon uptake. Meanwhile, TOMM20-positive EVs were found to be enriched in OA synovial fluid relative to controls [57]. In contrast, reparative EVs from MSCs, including those from umbilical cord, adipose tissue, and bone marrow sources, are shown to maintain chondrocyte homeostasis and alleviate OA pathology both *in vitro* and in preclinical *in vivo* models [[58], [59], [60], [61], [62]]. The dynamic balance between pathogenic and reparative EV subsets may play a central role in shaping the joint microenvironment, and shifts in their relative abundance can drive OA disease progression. Collectively, single-vesicle platforms are enabling precise stratification of EV subpopulations with diagnostic and pathological significance, offering new avenues for OA precision phenotyping, biomarker development, and targeted therapy.

### 2.3 Context-dependent EVs: cellular state governs EV function

**Fig. 1:**
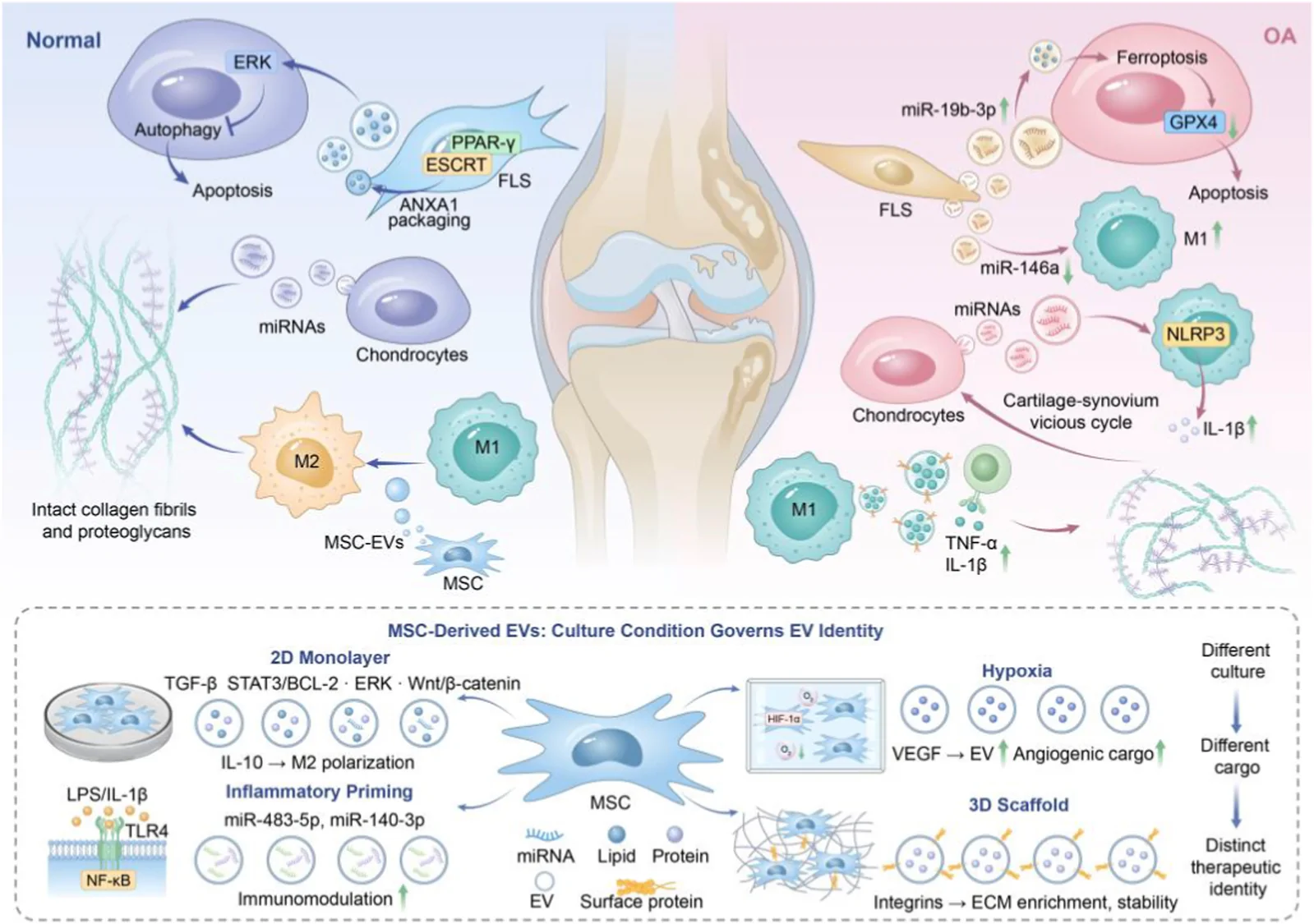
**State-dependent EV function in joint-resident cells and culture-condition-dependent EV identity in MSCs**. Top: EVs secreted by chondrocytes, synovial fibroblasts, and macrophages under homeostatic (blue, left) versus OA pathological (red, right) conditions. Key molecular cargo, downstream pathways, and functional consequences are indicated for each cell type. OA chondrocyte EVs activate macrophage NLRP3 inflammasome (IL-1β) and establish a cartilage–synovium vicious cycle; OA-FLS EVs (miR-19b-3p↑, miR-146a↓) induce chondrocyte ferroptosis and apoptosis, reversible by miR-146a overexpression; M1 macrophages sustain synovial inflammation, while M2 polarization supports tissue homeostasis. Bottom: A single MSC yields functionally distinct EVs under four culture conditions — 2D monolayer (basal anti-inflammatory, TGF-β/ERK/Wnt pathways), hypoxia (2–3× yield, pro-angiogenic, VEGF pathways), 3D scaffold (ECM-enriched, integrin-mediated, improved stability), and inflammatory priming (NF-κB-driven, miR-483-5p/miR-140-3p-enriched, enhanced immunomodulation). EVs, extracellular vesicles; OA, osteoarthritis; FLS, fibroblast-like synoviocytes; MSC, mesenchymal stromal cell.

The cargo sorting mechanisms elaborated above converge on a central biological principle: the same cell, in different functional states, can produce EVs with markedly distinct cargo composition and biological effects. In the OA joint, this applies to both disease-driving resident cells such as chondrocytes, synoviocytes, and immune cells, and to therapeutically administered MSCs. Whether the outcome is pathological deterioration or therapeutic enhancement, the underlying logic is consistent: altered cellular states reshape EV cargo, which in turn reprograms EV function. This dynamic relationship forms the conceptual foundation of context-dependent EV biology [63] (Fig. 1).

#### 2.3.1 Joint-resident cells: from homeostasis to pathological drive

Chondrocytes are a major source of EVs within the joint, and their EVs undergo functional alterations during OA progression [64]. Calcified EVs derived from mineral-containing autophagosomes released by chondrocytes have been found to initiate pathological cartilage calcification in early OA [65]. Senescent chondrocytes, which progressively accumulate in OA cartilage, produce pro-inflammatory factors packaged and released through EVs that contribute to continuous cartilage degeneration [66]. In the inflamed synovium, EVs from synovial fibroblasts undergo significant alterations during OA progression, where their key involvement in disease regulation has been confirmed through integrated clinical, animal, and public database analyses [4]. Furthermore, dysregulated fibroblast–macrophage interactions during OA-related synovitis are accompanied by increased exosome secretion from FLSs, implicating the role of EVs in amplifying synovial inflammation [67]. Macrophage-derived EVs are also shown to have a key role in OA pathogenesis [18]. Pro-inflammatory M1-like macrophages drive low-grade chronic inflammation in OA, while their secreted EVs deliver miR-155-5p to chondrocytes, inhibiting the GSK-3β/mTORC1-mediated autophagy axis and thereby accelerating cartilage degeneration [68].

#### 2.3.2 MSC-derived EVs: cellular state determines therapeutic potency

The therapeutic efficacy of MSC-derived EVs has been extensively validated across preclinical OA models, with demonstrated capacities to alleviate OA-associated pain, suppress inflammation, and promote cartilage regeneration [60,69,70]. However, the cellular source of EVs contributes to functional variability, whereby EVs derived from bone marrow, adipose tissue, and umbilical cord MSCs exhibit distinct properties, reflecting tissue-of-origin differences [71]. Moreover, a growing body of evidence suggests that even from the same cell source, EV cargo and function are not fixed properties but rather are highly dependent on the microenvironmental context of the parental MSCs [[72], [73], [74]]. These observations have direct translational implications for the manufacture and upscaling of EV-based therapeutics.

MSC-EVs derived under standard culture conditions already possess basal bioactivity, as MSCs naturally release functional growth factors, chemokines, and signaling molecules [75]. However, the environmentally responsive nature of MSCs means that microenvironmental changes profoundly modulate the MSC secretome, including both soluble factors and EVs [76]. For example, hypoxic preconditioning is among the most extensively investigated strategies for enhancing MSC-EV potency [77]. Combining hypoxia and inflammatory stimulation, which is designed to recapitulate the harsh microenvironment of joint injury sites, can further remodel the MSC secretome, where the HIF-1α signaling axis has been implicated in this process [78].

Inflammatory priming can exert biphasic effects on resultant MSC-EV function. Transient exposure to an inflammatory environment, such as by adding inflammatory cytokines to the culture medium, alters the cargo and functional properties of MSC-derived EVs, generally with beneficial outcomes due to enhanced anti-inflammatory, chondroprotective, and pro-repair effects [77]. However, while moderate inflammatory stimulation of MSCs can enhance the immunomodulatory effects of MSC-EVs, excessive or sustained stimulation can drive functional exhaustion, resulting in diametrically opposite secretory phenotypes depending on stimulus intensity [77,79].

In addition to biochemical conditioning through soluble factors, tailoring the biophysical culture environment of MSCs can also enhance the therapeutic functions of their EVs in the context of joint repair [80]. Compared to standard 2D monolayer culture, 3D culture systems more faithfully recapitulate *in vivo* physiological conditions. For example, 3D spheroid culture has been shown to significantly enhance MSC survival, stemness, immunomodulatory function, and EV therapeutic potential compared to 2D culture [81]. Advanced 3D dynamic culture systems, including one that incorporated TGF-β3 [82], have also been developed to improve EV yield and optimize therapeutic performance. The 3D microenvironment regulates MSC fate and EV function partly through mechanosensitive signaling pathways [83].

As visualized in Fig. 1, the bidirectional shift of EV bioactivity is exemplified by both homeostatic and pathological EV phenotypes from joint-resident cells, alongside divergent MSC-EV profiles shaped by distinct culture niches. Under normal physiological conditions, FLS package Annexin A1 (ANXA1) into EVs via the PPARγ/ESCRT axis; upon internalization by chondrocytes, ANXA1 inhibits ERK phosphorylation and activates autophagy, thereby preserving cartilage homeostasis [84]. In the OA microenvironment, however, IL-1β-stimulated FLS secrete EVs enriched in miR-19b-3p, which induce chondrocyte ferroptosis (GPX4 downregulation) and apoptosis while sustaining M1 macrophage polarization, thereby amplifying inflammatory cascades [85]. Notably, endogenous miR-146a is downregulated in OA-FLS, and overexpression of miR-146a in FLS-EVs reverses these pathological effects by promoting M1-to-M2 repolarization of synovial macrophages [86]. Concurrently, OA chondrocyte-derived exosome-like vesicles activate the macrophage NLRP3 inflammasome, driving mature IL-1β secretion and establishing a self-perpetuating cartilage–synovium inflammatory cycle [87]. The M1/M2 macrophage imbalance constitutes a central pathogenic mechanism in OA chronic low-grade inflammation [88]: M1 macrophages drive synovial inflammation through antigen presentation and cytokine signaling (TNF-α, IL-6), whereas MSC-derived EVs can reprogram M1 to M2, restoring immune microenvironment homeostasis [67].

Importantly, the principle that EV function is governed by the state of the parent cell extends to therapeutic MSCs. As shown in the lower panel, identical MSCs yield functionally divergent EVs depending on culture conditions. Under conventional 2D monolayer culture, MSCs maintain basal secretory activity through TGF-β/STAT3/BCL-2, Raf/MEK/ERK, and Wnt/β-catenin pathways; the resulting EVs (containing miR-27a-3p and other cargo) promote M2 macrophage polarization via IL-10 secretion, exerting basal anti-inflammatory functions [[89], [90], [91]]. Hypoxic culture (1–3% O_2_) markedly enhances pro-angiogenic capacity and increases EV yield 2–3 fold, activating VEGF receptor-mediated downstream pathways in recipient cells, although the underlying molecular mechanisms remain incompletely defined [78,92,93]. In 3D scaffold culture, integrin-mediated cell–matrix interactions (e.g., RGD-coupled hydrogels modulating stress relaxation) better recapitulate the *in vivo* niche; the resulting EVs are enriched in ECM-associated proteins and exhibit improved functional stability, though mechanistic details remain poorly understood [89,94]. Inflammatory priming (e.g., LPS stimulation) activates NF-κB signaling, significantly altering EV cargo — miR-483-5p and miR-140-3p are enriched, endowing EVs with enhanced immunomodulatory potency [93,95]. Together, these comparisons underscore the core concept that culture conditions govern EV identity and therapeutic function, providing an experimental rationale for optimizing MSC-EV-based treatment strategies.

#### 2.3.3 Experimental validation and translational implications

The evidence above, generated from both OA-resident joint cells and therapeutic MSCs, indicates that EV function is not a fixed property determined solely by cellular origin, but rather emerges from the interplay between the parent cell phenotype, microenvironmental cues, activation state, and disease stage. This has important implications for both mechanistic studies and translational development in OA research.

Experimental variables that influence EV composition and activity, including source cell treatment, culture conditions, inflammatory status, and disease stage, should be rigorously defined and fully documented. Cell type alone is insufficient for cross-study comparisons, as EV cargo and function can vary substantially in response to changes in the cellular environment. Standardized reporting of these parameters will be essential for improving reproducibility and interpretation across the field.

These emerging findings also challenge the common binary classification of EVs as either “pathogenic” or “therapeutic”. EVs released under pathological conditions should not be assumed to be uniformly harmful, as they may also mediate adaptive, compensatory, or tissue-protective responses. Conversely, EVs from therapeutic cell sources are not inherently beneficial under all conditions, since their molecular cargo and biological activity are highly dependent on the physiological state of the parent cells and manufacturing conditions. A more informative framework is therefore to view EVs as context-dependent biological effectors whose function reflects the state of the cells and tissues from which they originate.

A major translational priority is the development of robust classification frameworks that define EV populations according to molecular composition and biological activity rather than source alone. Such frameworks should integrate EV surface markers, cargo profiles (e.g., miRNAs, proteins, lipids, mitochondrial DNA, and damage-associated molecular patterns (DAMPs), and validated biological effects on recipient cells. This context-dependent perspective is equally important for biomarker development and therapeutic translation [96,97]. EV cargo may provide a dynamic readout of the local joint microenvironment and ongoing pathological processes, potentially offering greater biological resolution than conventional measures of overall disease severity. Meanwhile, a deeper understanding of how environmental cues influence EV biogenesis may enable the rational engineering of MSC-EVs with more consistent and defined therapeutic properties.

To facilitate navigation of the expanding literature on EV-mediated intercellular communication in OA, Supplementary Table 1 provides a structured summary of the major EV populations discussed in this review, catalogued by cellular source, molecular cargo, target cells, functional effects, and OA-related mechanisms. Consistent with the principle of context-dependent EV function elaborated above, the table highlights how the same cell type can give rise to both homeostatic and pathogenic EV phenotypes—and how therapeutic MSC-EVs can be rationally engineered to favour reparative outcomes. The following sections examine these EV populations in mechanistic detail, beginning with the role of endogenous joint-resident EVs in OA pathogenesis.

## 3 Role of EVs in OA pathogenesis

### 3.1 Effects of EVs on chondrocytes

**Fig. 2:**
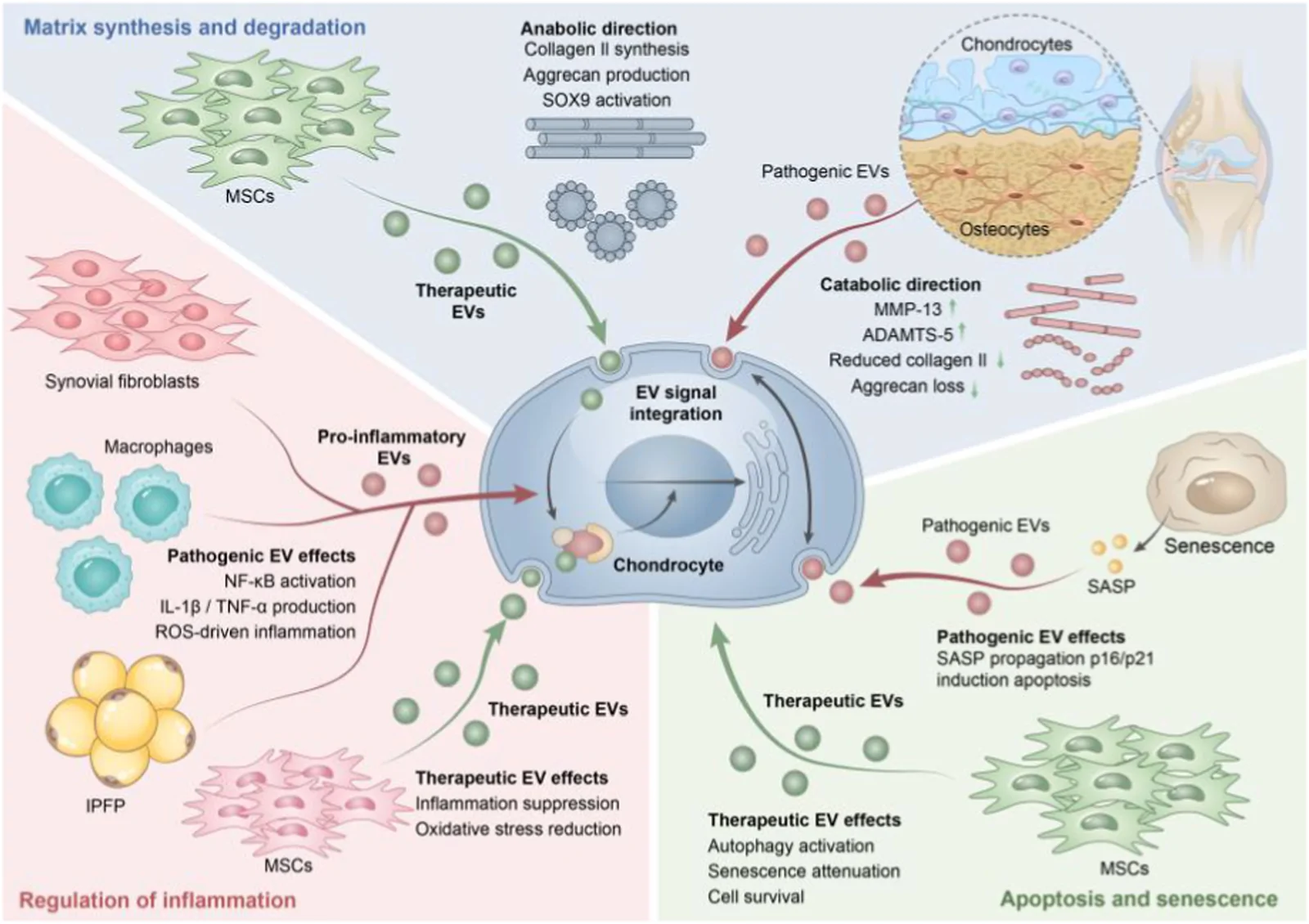
Context-dependent integration of EV signaling in chondrocyte fate and cartilage homeostasis.

Chondrocytes, as the sole cellular residents of articular cartilage, function as central integrators of EV-mediated signals within the OA joint microenvironment (Fig. 2). Their phenotypic stability, metabolic activity, and survival are dynamically shaped by EV cargo originating from diverse joint tissues, including subchondral bone, synovium, infrapatellar fat pad, and immune cells. Chondrocyte responses to EVs are highly heterogeneous and are influenced by EV source, molecular cargo (e.g., miRNAs, proteins, lipids), disease stage, and the local inflammatory or oxidative milieu (Fig. 2). Consequently, EV-mediated communication can differentially regulate key processes involved in OA pathogenesis, including inflammatory signaling, ECM homeostasis, and cell fate decisions. This section examines the mechanisms through which EVs influence these three fundamental aspects of chondrocyte biology that are implicated in OA progression.

#### 3.1.1 Regulation of inflammation

EVs serve as potent vectors for inflammatory reprogramming of chondrocytes. Pro-inflammatory EVs derived from activated synovial fibroblasts, macrophages, or osteoarthritic infrapatellar fat pad (IPFP) deliver cargo that amplifies NF-κB signaling and cytokine production (Fig. 2). For instance, EVs from lipopolysaccharide (LPS)-stimulated macrophages induce a pronounced pro-inflammatory response in chondrocytes, promoting IL-1β and TNF-α secretion and accelerating cartilage degradation in murine models [68]. Synovial fibroblast-derived small EVs (sEVs) from OA patients transfer inflammatory mediators to chondrocytes, exacerbating synovitis and cartilage catabolism [4,98]. IPFP-derived sEVs from OA patients similarly propagate inflammation, whereas inhibiting their release through GW4869 is shown to attenuate inflammatory cascades in chondrocytes [99]. Conversely, EVs from regenerative cell sources exert anti-inflammatory effects. MSC-derived EVs, particularly those from umbilical cord (UC) or Wharton's jelly (WJ), suppress chondrocyte inflammatory pathways by delivering miRNAs or proteins that downregulate IL-6, IL-8, and MMP expression [59,100,101]. EVs from adipose tissue (AD) MSCs are shown to mitigate oxidative stress in OA chondrocytes by modulating NOX4-related pathways, thereby reducing ROS-driven inflammation [102] (Fig. 2). Neutrophil-derived EVs also contribute to chondroprotection by promoting proteoglycan synthesis and reducing catabolic expression, through upregulating frizzled-related protein 5 (SFRP5) in chondrocytes [103]. EVs from joint-related cell sources hence act as bidirectional regulators in OA, whereby pathogenic EVs from inflamed tissues drive a destructive loop, while therapeutic EVs from preconditioned or engineered MSCs can help restore immunological balance [104].

#### 3.1.2 Matrix synthesis and degradation

EVs critically influence the anabolic-catabolic equilibrium of cartilage ECM (Fig. 2). Pathogenic EVs disrupt homeostasis by inducing the secretion of catabolic enzymes and suppressing matrix synthesis in articular cartilage. For instance, osteocyte-derived EVs in subchondral bone carry miR-23b-3p, which targets OTUD4 in chondrocytes, impairing mitophagy and shifting cells toward catabolic behavior, evidenced by elevated MMP-13 and ADAMTS-5 together with reduced collagen type II and aggrecan [15]. OA chondrocyte-derived sEVs enriched in connexin 43 (Cx43) propagate SASP factors (e.g., IL-1β), further driving ECM degradation across joint tissues [105]. In contrast, regenerative EVs promote matrix preservation and synthesis (Fig. 2). UC-MSC-derived EVs have been shown to enhance collagen deposition, autophagy, and M2 macrophage polarization, collectively protecting cartilage integrity [105]. sEVs from WJ-MSCs can also stimulate chondrocyte migration, proliferation, and ECM production through SOX9-mediated pathways [101,106]. The therapeutic effects of MSC-EVs can be amplified by purposeful modification of EV cargo. For example, curcumin-loaded sEVs derived from AD-MSCs simultaneously suppress MMPs and boost aggrecan synthesis, while EVs functionalized with antisense oligonucleotides against MMP-13 achieve chondrocyte-targeted inhibition of matrix degradation [107]. Notably, due to their small size, EV-mediated delivery of WNT3a can overcome cartilage matrix barriers to activate regenerative signaling in cells [108]. These findings position EVs as dynamic modulators of chondrocyte phenotype, which are capable of either reinforcing tissue degeneration in OA or reinstating reparative states depending on EV cargo and source [9,109].

#### 3.1.3 Apoptosis and senescence

EVs significantly impact chondrocyte viability and senescence, processes central to chronic OA progression (Fig. 2). Senescent chondrocytes accumulate in OA joints, secreting SASP factors that drive senescence and matrix destruction in a paracrine manner [66,70]. OA chondrocyte-derived sEVs serve as important vehicles for senescence propagation, transferring SASP components (e.g., IL-1β) and senescence-inducing miRNAs to neighboring chondrocytes, synoviocytes, and bone cells, hence amplifying joint-wide tissue degeneration [70,105] (Fig. 2) Conversely, MSC-sEVs enhance chondrocyte survival by upregulating autophagy (through BCL2-Beclin-1 axis modulation) and suppressing apoptosis [110]. EVs from UC-MSCs endowed with chondrocyte-targeting capability can rejuvenate senescent chondrocytes by restoring metabolic function and reducing p16/p21 expression [106,111]. CircRNA-modified EVs (e.g., by circCREBBP knockdown) can also attenuate senescence by promoting TGF-β2 signaling and ECM anabolism. Hence, EVs can play dual roles in the stress-response network of chondrocytes, where they may drive the propagation of cellular senescence under pathological conditions such as OA but also enable tissue rejuvenation when engineered to amplify therapeutic properties [70,112].

The effects of EVs on chondrocytes are intrinsically interconnected, as individual EV cargo molecules frequently influence multiple cellular pathways. For example, pro-inflammatory EV cargo (e.g., miR-23b-3p) simultaneously triggers catabolism, suppresses matrix synthesis, and accelerates senescence [15]. Meanwhile, anti-inflammatory MSC-EVs concurrently enhance autophagy, reduce apoptosis, and stimulate anabolic responses [59,101,113]. This pleiotropic activity reflects the integrated nature of chondrocyte responses, in which inflammatory signaling, matrix homeostasis, and cell fate regulation are linked through dynamic and reciprocal feedback networks. Consequently, modulation of a single pathway can produce coordinated effects across multiple aspects of OA pathology. For example, EV-mediated suppression of oxidative stress (through NOX4 regulation) concurrently dampens inflammation, preserves ECM, and mitigates ferroptosis [102]. Conversely, senescence-propagating EVs from IPFP or synovium can establish self-reinforcing pathogenic feedback loops that perpetuate inflammation and matrix breakdown [4,99].

Recognizing the context-dependent nature of EV-mediated signaling is essential for therapeutic design. Strategies leveraging EV engineering (e.g., miRNA loading, surface modification) or preconditioning of the source cells (e.g., cytokine priming, nanosecond pulsed fields) aim to tip the balance towards joint homeostasis instead of OA progression [100,114]. It should be noted that much of the current evidence for the chondrocyte-regulatory effects of EVs is derived from *in vitro* chondrocyte cultures and rodent OA models, while studies using primary human OA chondrocytes or investigating EVs from clinical biofluid samples (e.g., synovial fluid) remain comparatively scarce. Prospective validation in human cohorts is needed to establish the physiological relevance of EV-mediated mechanistic pathways.

Chondrocytes integrate EV-derived signals from multiple joint-resident cell types, including synovial fibroblasts, macrophages, and IPFP-derived MSCs, to regulate inflammatory responses, matrix turnover, and cell survival. EV inputs are not binary but exert effects on the target cell in a context-dependent manner, determined by EV cargo composition, the activation state of the recipient chondrocyte, and the surrounding joint microenvironment. Pathogenic EVs promote NF-κB activation, pro-inflammatory cytokine production, oxidative stress, and catabolic programmes, leading to increased expression of matrix-degrading enzymes such as MMP-13 and ADAMTS-5, aggrecan loss, SASP propagation, and apoptosis. In contrast, protective EVs support anabolic signaling, including SOX9 activation, collagen II and aggrecan synthesis, autophagy induction, oxidative stress reduction, and metabolic restoration, thereby enhancing chondrocyte survival and matrix integrity. The net outcome reflects the dynamic balance between converging anabolic and catabolic EV signals rather than the action of individual EV populations, providing a conceptual framework for understanding how EV cargo heterogeneity and joint context shape cartilage degeneration or repair in OA.

### 3.2 EVs in synovial inflammation and immune regulation

**Fig. 3:**
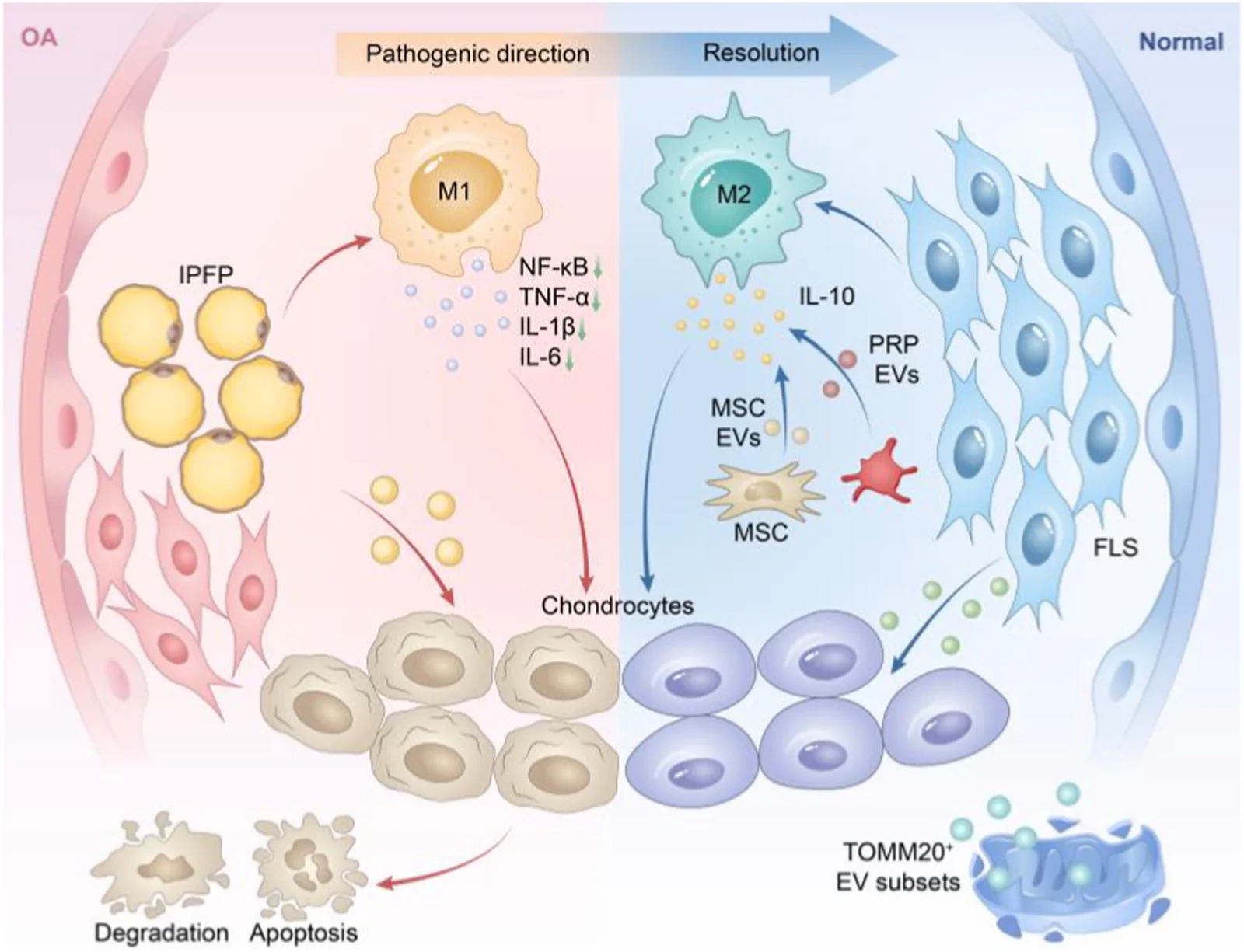
Bidirectional immunoregulatory roles of EVs in the synovial microenvironment during OA progression and resolution.

The synovium functions as a dynamic immunoregulatory interface within the OA joint, where low-grade chronic inflammation mainly involving the synovium is one of the hallmarks of OA, driving tissue catabolism and pain sensitization. EVs are key mediators of stromal–immune crosstalk, actively shaping the synovial microenvironment through context-dependent signaling rather than passive inflammatory amplification (Fig. 3). Emerging evidence positions EVs as bidirectional regulators that integrate mechanical, metabolic, and inflammatory cues to modulate immune cell recruitment, activation states, and resolution pathways in the synovium, thereby influencing cartilage integrity and subchondral bone remodeling across the whole joint [1,3,11] (Fig. 3).

#### 3.2.1 Synoviocyte-derived EVs

FLSs release EVs whose cargo profiles dynamically reflect disease stage, inflammatory activation, and biomechanical stress (Fig. 3). In OA, FLS-derived sEVs exhibit altered miRNA signatures that propagate inflammatory signals to chondrocytes, inducing catabolic enzyme expression and suppressing ECM synthesis [4,67,98]. Notably, synovial inflammation correlates with increased EV secretion from activated FLSs, which transfer pro-inflammatory mediators to neighboring macrophages and chondrocytes, establishing a feed-forward loop of joint degeneration [4,67]. Conversely, synovial MSC-derived EVs have demonstrated protective capacity, whereby LPS-preconditioned synovial MSC-EVs enhance trophic support and mitigate cartilage damage through immunomodulatory cargo [104]. Furthermore, synovial fluid EV composition shifts with OA progression, for instance, the observation that TOMM20^+^ EV subsets are elevated in OA synovial fluid versus trauma controls suggests potential use of mitochondrial stress signatures embedded within EVs as disease-stage biomarkers [57,104] (Fig. 3). Importantly, EV-mediated communication between synoviocytes and chondrocytes is bidirectional. While synoviocyte-derived EVs influence chondrocyte behavior, chondrocyte-derived EVs can also modulate synovial fibroblast activation, highlighting the reciprocal and interconnected nature of joint tissue crosstalk [105].

#### 3.2.2 Macrophage polarization

EVs are key regulators of macrophage functional plasticity within the synovium, influencing context-dependent transitions between and beyond the M1/M2 phenotypes. Platelet-rich plasma (PRP)-derived EVs have been shown to suppress pro-inflammatory activation of synovial macrophages while promoting reparative functions, contributing to reduced synovitis and cartilage protection in preclinical models [115]. UC-MSC-EVs have been shown to drive M2-like macrophage polarization, enhancing anti-inflammatory cytokine secretion (e.g., IL-10) and facilitating inflammation resolution [59,113]. Neutrophil-derived EVs contribute to joint tissue homeostasis by promoting proteoglycan synthesis and attenuating synovial inflammation [103]. However, the effects of EVs on macrophages are highly dependent on their cellular source and biological state. EVs derived from inflammatory macrophages exacerbate chondrocyte apoptosis and matrix degradation, whereas IPFP-MSC-EVs have been reported to accelerate OA progression, potentially through poorly defined pathogenic EV subpopulations [57]. These observations further highlight the context-dependent nature of EV-mediated signaling within the OA joint.

The therapeutic potential of targeting macrophage plasticity is illustrated by engineered EV approaches, such as miR-27b-3p-enriched sEVs targeting the CSF-1 axis to selectively reprogram macrophage responses and restore joint homeostasis. Collectively, these findings point to a central role of synovial macrophages in linking immune dynamics to cartilage health, as macrophage polarization states govern the production of catabolic and inflammatory mediators that directly affect chondrocyte survival, matrix integrity, and disease progression [18,116].

#### 3.2.3 Cytokine and chemokine signaling

In OA, synovial fluid EVs carry surface-bound cytokines and chemokines that can potentiate leukocyte recruitment and amplify synovial inflammation [117]. Interestingly, EVs can also exert counter-regulatory effects, exemplified by adenosine-generating EVs in synovial fluid that suppress T-cell activation independently of regulatory T-cells [118]. MSC-EVs further modulate inflammatory signaling by inhibiting NF-κB signaling in macrophages, thereby downregulating TNF-α, IL-1β, and IL-6 while upregulating IL-10 [19,113,119]. In parallel, adipose MSC-EVs mitigate oxidative stress in chondrocytes through anti-inflammatory and anti-catabolic cargo. These examples further illustrate that EV-mediated cytokine regulation is context-dependent and functionally bidirectional, whereby EVs from endogenous OA joint-resident cells often reinforce inflammatory signaling. In contrast, therapeutically administered MSC-EVs tend to promote inflammatory resolution [59,113]. Moreover, EVs contribute to spatial coordination of joint inflammation, as platelet-derived EVs can traverse tissue barriers to deliver immunomodulatory signals into the synovial fluid, while macrophage-derived EVs participate in inter-tissue communication, including through strategies to enable targeted drug delivery to subchondral bone [14].

EV-mediated communication hence forms a dynamic, adaptive network linking synovial stroma, immune cells, cartilage, and bone. Reciprocal feedback loops are evident within this system: synovitis-associated FLSs release EVs that activate macrophages, whose subsequent EV outputs further modulate chondrocyte metabolism and osteoblast activity [4]. This bidirectional exchange positions EVs as important regulatory nodes in OA pathogenesis, both reflecting the biochemical state of the joint microenvironment (e.g., oxidative stress, SASP) and actively shaping disease trajectory [112,120].

Contextual variables including donor cell type, preconditioning stimuli (e.g., LPS, hypoxia), and recipient cell state collectively dictate whether EV signaling promotes resolution or disease amplification [104]. Added complexity arises from EV subtype heterogeneity, as exosomes, microvesicles, and apoptotic bodies can exert distinct and sometimes non-overlapping biological functions. For instance, osteoclast-derived microvesicles have been shown to exert potent pro-chondrogenic, pro-osteogenic, and anti-inflammatory functions, which were unique to osteoclast microvesicles rather than their exosomes or apoptotic bodies, and were not seen in the microvesicles of other cell types [121]. Taken together, EVs should be viewed not as isolated intercellular messengers but as embedded components of a whole-joint regulatory system. To advance precision intervention in OA, future efforts should prioritize the systematic definition of EV subpopulation-specific functions and the contextual determinants that govern their immunomodulatory activity [1,57].

EVs function as dynamic mediators of stromal–immune crosstalk, actively shaping the joint microenvironment along a continuum from pathogenic degradation to inflammatory resolution. Left: In the OA microenvironment, EVs derived from specific joint-resident cells, such as those from the IPFP or pro-inflammatory M1 macrophages, exacerbate disease progression by driving chondrocyte apoptosis and extracellular matrix degradation. Middle to Right: The transition toward tissue resolution is driven by immunomodulatory EVs, such as those derived from MSCs and PRP. These therapeutic EVs promote the phenotypic transition of macrophages from an M1 to an anti-inflammatory M2 state, characterized by upregulated IL-10 secretion. Furthermore, MSC-EVs mediate the downregulation of pro-inflammatory cytokines (TNF-α, IL-1β, and IL-6) via the inhibition of NF-κB signaling. In a healthy state, FLSs maintain reciprocal, homeostatic EV communication with chondrocytes to preserve joint integrity. Bottom right: Distinct EV subsets, notably TOMM20^+^ EVs reflecting intracellular mitochondrial stress, are enriched in the synovial fluid during disease pathogenesis, highlighting their potential utility as biomarkers for OA staging.

### 3.3 EVs and subchondral bone remodeling

**Fig. 4:**
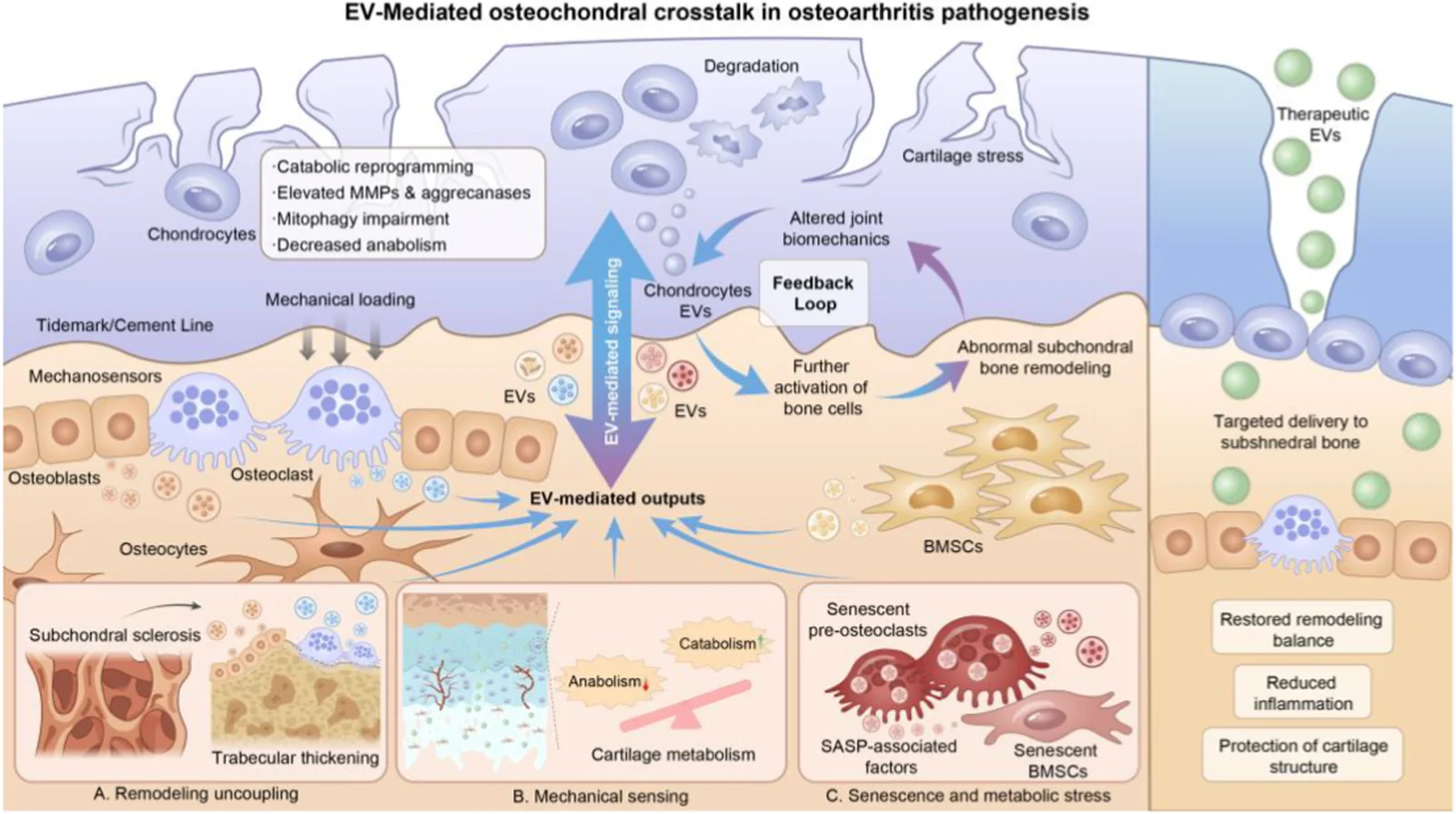
EV-mediated osteochondral crosstalk in OA.

Subchondral bone is increasingly recognized for its function beyond a passive structural scaffold as a dynamic, metabolically active tissue that directly participates in OA pathogenesis. Aberrant remodeling of subchondral bone, including sclerosis, cyst formation, trabecular thickening, and altered vascularization often precedes overt cartilage degeneration and correlates strongly with pain and OA disease progression [15,122,123]. This remodeling reflects dysregulated coupling between bone formation and resorption, driven by pathological intercellular signaling within the osteochondral unit (Fig. 4). EVs have been implicated as key mediators of this communication, facilitating bidirectional crosstalk among osteoblasts (OBs), osteoclasts (OCs), osteocytes, chondrocytes, and immune cells. EV cargo, encompassing various molecular species such as miRNAs, proteins, lipids, and metabolites collectively integrates mechanical, inflammatory, and metabolic cues to modulate cellular behavior across joint tissues [9,11,124,125].

#### 3.3.1 Osteoblast–osteoclast communication

Within subchondral bone, EVs orchestrate the delicate balance between bone formation and resorption (Fig. 4). Osteoblast-derived EVs regulate osteoclast differentiation and activity through RANKL mechanisms [124]. Meanwhile, osteoclast-derived microvesicles exhibit osteoanabolic properties, directly influencing local bone formation and OC-mediated remodeling [121]. Conversely, preosteoclasts in metabolic syndrome-associated OA undergo senescence during early disease, acquiring a unique secretome that activates the COX2-PGE2 axis to stimulate OB progenitor differentiation while suppressing OC maturation [126]. This EV-mediated shift leads to pathological thickening of the subchondral bone plate and trabeculae prior to the onset of cartilage damage (Fig. 4). Mechanical stress further amplifies the dysregulation of bone remodeling in OA, with EVs acting as key messengers in this process. Osteocytes, as primary mechanosensing cells in the subchondral bone, release EVs enriched in miR-23b-3p under pathological loading, which reflects altered bone-cell signaling and disrupts chondrocyte homeostasis [15]. Meanwhile, senescent bone marrow MSCs in subchondral bone contribute to sclerosis through the release of SASP factors packaged into EVs, which impair microenvironmental homeostasis [127,128] (Fig. 4). EV cargo also dynamically encodes the biochemical state of the bone niche, for example, elevated PGE2 in subchondral bone drives sensory nerve ingrowth and structural deterioration through EP4 receptor signaling, a pathway modifiable by COX2 inhibition or targeted EV delivery [129]. Collectively, these observations position EVs as both real-time indicators and active mediators of bone-cell coupling, whereby disease-associated shifts in EV composition contribute directly to uncoupled remodeling processes characteristic of OA [121,124,126,127].

#### 3.3.2 Crosstalk between cartilage and bone via EVs

EVs establish a continuous molecular dialogue between cartilage and subchondral bone, embedding osteochondral crosstalk within the whole-joint framework of OA (Fig. 4). Bone-derived EVs can directly modulate chondrocyte function. For example, osteocyte-secreted EVs enriched in miR-23b-3p suppress OTUD4 in chondrocytes, impairing mitophagy and thereby promoting catabolic matrix degradation while inhibiting anabolic activity [15] (Fig. 4). Similarly, subchondral bone EVs containing miR-210-3p modulate angiogenesis, where reduced levels are associated with excessive vascular invasion that disrupts bone-cartilage interface integrity and accelerates cartilage loss. Cartilage and synovium derived EVs can reciprocally influence subchondral bone remodeling (Fig. 4). FLS-derived EVs exhibit altered protein and miRNA profiles in OA, promoting inflammatory responses in chondrocytes and potentially modulating the activity of OBs and OCs [4].

In contrast, therapeutic EV populations including bone marrow MSC-EVs, UC-MSC-EVs, and engineered MSC-EVs are shown to suppress synovial inflammation, reduce chondrocyte apoptosis, promote M2 macrophage polarization, and attenuate subchondral bone deterioration in preclinical OA models [119,130]. Emerging strategies enabling targeted EV delivery to subchondral bone, such as by using macrophage-derived EVs functionalized with targeting peptides or ST-needle hydrogel systems, further enhance EV retention and therapeutic efficacy against pathological bone remodeling.

These advances underscore EVs as spatial and temporal coordinators of osteochondral homeostasis. Disruption of this communication network creates self-amplifying pathological loops, in which abnormal subchondral bone remodeling alters joint biomechanics and intensifies mechanical stress on cartilage, while cartilage-derived inflammatory EVs further activate subchondral bone cells (Fig. 4). In parallel, vascular and sensory nerve invasion, partly mediated by osteoclast-derived factors such as SLIT3, links structural remodeling to pain signaling [131]. Longitudinal analyses confirm that early subchondral alterations precede and predict subsequent cartilage degeneration, positioning EV-mediated crosstalk as a temporal driver of OA progression [122].

Collectively, EVs function as critical conduits of intercellular communication within the subchondral bone microenvironment and across the osteochondral interface (Fig. 4). They translate physiological cues arising from mechanical strain, inflammation, senescence, and metabolic stress into coordinated, or dysregulated tissue responses. In OA, pathological EV signaling drives uncoupled bone remodeling and propagates damage bidirectionally between bone and cartilage, reinforcing disease progression as a whole-joint disorder. Conversely, the same system presents therapeutic opportunities, whereby harnessing EVs for targeted delivery or leveraging their regenerative cargo offers the potential to restore osteochondral homeostasis. Future work should focus on delineating cell-type-specific EV signatures, defining EV cargo dynamics across OA stages, and optimizing engineered EV platforms to disrupt maladaptive crosstalk while preserving physiological communication [3,11,16,132,133]. These approaches will be essential for developing disease-modifying EV interventions that address the integrated pathophysiology of OA.

EVs mediate bidirectional communication between articular cartilage and subchondral bone, driving dysregulated remodeling of the osteochondral unit in OA. In cartilage, chondrocytes undergo a catabolic shift marked by increased matrix-degrading enzymes, impaired mitophagy, and reduced anabolic activity, leading to ECM breakdown and altered joint biomechanics. In subchondral bone, EVs released by osteoblasts, osteoclasts, osteocytes, and bone marrow stromal cells integrate mechanical loading, metabolic stress, and senescence-associated cues, contributing to disrupted osteoblast–osteoclast coupling, subchondral sclerosis, and trabecular thickening in OA. Osteocytes act as mechanosensors that modulate load-dependent EV release, whereas senescent bone cells secrete EVs enriched in SASP-associated factors, further amplifying inflammatory and remodeling pathways. EV-mediated signaling establishes self-reinforcing feedback loops between bone and cartilage, propagating degeneration across the osteochondral interface. In contrast, EVs can also play an important therapeutic role in OA, whereby regenerative or engineered EVs targeting subchondral bone may rebalance remodeling, attenuate inflammation, and help preserve cartilage integrity.

## 4 Diagnostic and prognostic value of EVs in OA

OA lacks robust biomarkers for early diagnosis, prediction of structural progression, and stratification of its heterogeneous disease phenotypes. Current biomarkers include serum COMP, hyaluronic acid, and urinary CTX-II, but these offer limited sensitivity, specificity, and mechanistic insight into whole-joint pathophysiology [134,135]. EVs represent a biologically informative alternative because they encapsulate molecular cargo reflective of their parental cells, the evolving joint microenvironment, and systemic influences, thereby integrating biological signals across cartilage, synovium, subchondral bone, and IPFP [1,4]. Importantly, EVs are active mediators of OA pathogenesis rather than passive by-products, making their cargo dynamic indicators of disease activity instead of static correlates.

**Fig. 5:**
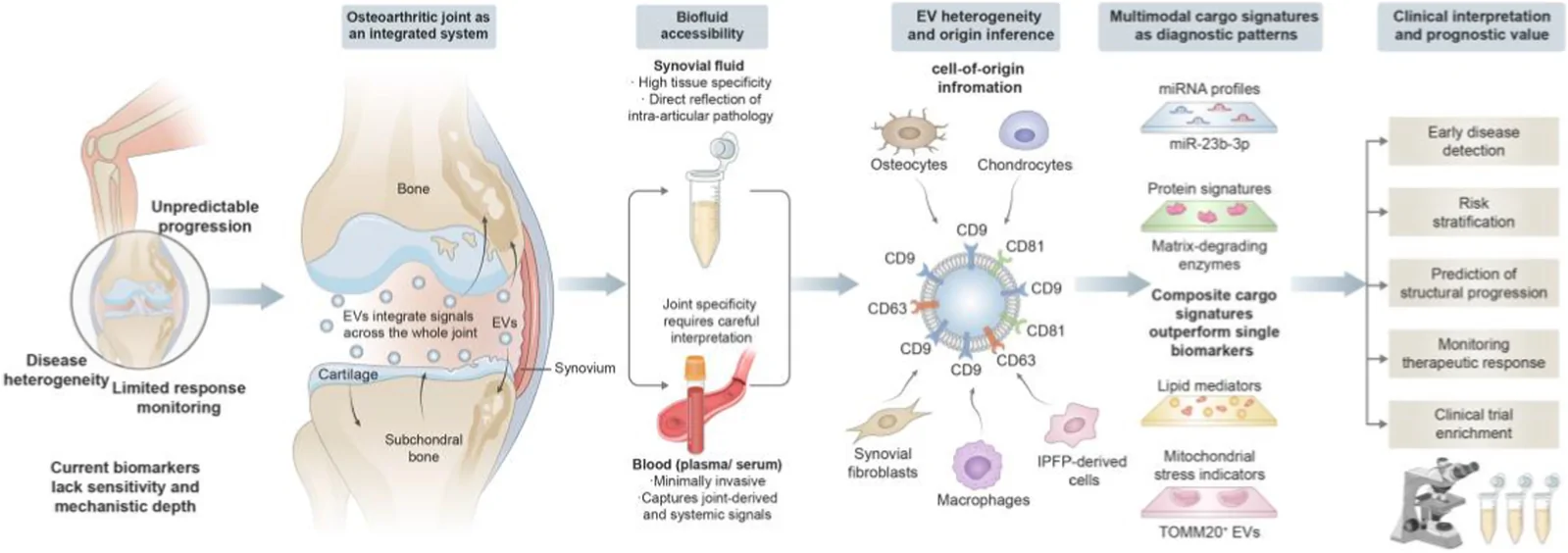
EV-based multimodal biomarkers enable joint-informed diagnosis and stratification in OA.

EVs isolated from synovial fluid provide tissue-proximal snapshots of intra-articular pathology. For example, synovial fluid EVs enriched for TOMM20, a mitochondrial marker, are elevated in OA patients compared with trauma controls, consistent with disrupted mitochondrial homeostasis in the OA joint [57]. IFCM-based EV profiling in native synovial fluid has shown that CD82^+^ and CD9^+^ EVs can discriminate septic from aseptic prosthetic joint loosening with high diagnostic accuracy [136]. Likewise, synovial fibroblast-derived small EVs carry distinct miRNA profiles that modulate inflammatory responses in chondrocytes, linking specific EV subpopulations to synovitis severity [67]. Osteocyte-derived EVs containing miR-23b-3p influence chondrocytes and can promote OA progression, illustrating how subchondral bone-derived cargo contributes to cartilage catabolism through osteochondral crosstalk [15]. Meanwhile, circulating EVs in plasma or serum enable minimally invasive disease monitoring (Fig. 5). High-resolution flow cytometry of plasma EVs has identified altered surface marker expression and cytokine cargo across different EV subsets that correlate with radiographic progression of knee OA [117].

EV-based biomarkers offer several key advantages, including the potential for multimodal cargo interrogation (such as proteins, nucleic acids, lipids, and metabolites), inherent stability of EVs in biofluids and cargo protection from enzymatic degradation, and the possibility to infer cellular origin through characteristic surface markers [137,138] (Fig. 5). Unlike soluble biomarkers diluted in the circulation, EVs concentrate disease-relevant signals that can reveal pathogenic intercellular communication networks, such as macrophage–chondrocyte signaling mediated by EV-associated miRNAs [3]. However, substantial barriers remain in the clinical translation of EV-based biomarkers. Extensive heterogeneity in EV size, biogenesis, and cellular origin complicates analytical standardization [139,140]. Methodological variability in EV isolation, characterization, and molecular profiling leads to inconsistent findings across studies [139,140]. Moreover, most evidence is derived from small cross-sectional cohorts, while robust longitudinal validation across diverse OA endotypes (e.g., metabolic OA) remains limited [117]. Distinguishing pathogenic from homeostatic EV populations also remains challenging because endogenous EVs released by diseased joint tissues can exacerbate OA, whereas therapeutically administered EVs exert regenerative effects [141].

From a translational perspective, EV signatures may prove most valuable for patient stratification in clinical trials, identifying individuals at risk of rapid progression, and for monitoring responses to disease-modifying therapies through longitudinal cargo profiling [134,142]. However, care should be taken not to overstate the clinical readiness of EVs, as no EV biomarker has yet achieved rigorous analytical validation under OARSI or FDA guidelines [137,143]. Future efforts require standardized protocols consistent with MISEV recommendations [33], large prospective multicenter cohorts, and integration with imaging and clinical endpoints [117,139,143]. Until then, EVs should be regarded as promising investigational biomarkers that provide unique insight into OA pathobiology while highlighting the challenges of translating complex intercellular communication networks into clinically actionable diagnostics (Fig. 5).

OA involves coordinated pathological changes across cartilage, synovium, subchondral bone, and immune compartments, but current diagnostic approaches are limited by late detection, poor sensitivity, and insufficient mechanistic insight. EVs act as integrative reporters of joint biology by capturing context-dependent signals from multiple joint-resident cell types, including chondrocytes, synovial fibroblasts, macrophages, and osteocytes. EVs are accessible in clinically relevant biofluids, with synovial fluid EVs providing high joint specificity and blood (plasma or serum) EVs offering minimally invasive access to both joint-derived and systemic information. Heterogeneous EV populations, defined by size and surface markers (such as CD9, CD63, CD81, and CD61), enable inference of cellular origin and pathological context. Multimodal EV cargo signatures, including miRNA profiles, protein signatures associated with matrix remodeling, lipid mediators, and indicators of mitochondrial stress, provide composite diagnostic patterns that outperform single-analyte biomarkers. Integration of these EV-derived signals supports early disease detection, patient stratification, prediction of structural progression, monitoring of therapeutic response, and informed design of clinical trials. Despite their promise, translation of EV-based diagnostics requires careful interpretation of biofluid origin, rigorous standardization of EV isolation and analysis (according to MISEV recommendations), longitudinal validation and regulatory approval. Together, EV-based biomarkers represent a dynamic and mechanistically informed approach to OA diagnosis that reflects whole-joint pathology rather than isolated tissue changes.

## 5 Therapeutic potential of EVs in OA

### 5.1 Preclinical studies of MSC-derived EVs

Robust preclinical evidence supports the therapeutic efficacy of MSC-EVs across diverse OA models [3,11]. For example, intra-articular delivery of human UC-MSC-EVs has been shown to significantly reduce cartilage degradation, synovial inflammation, and chondrocyte apoptosis while promoting ECM anabolism and M2 macrophage infiltration in rat and mouse OA models [59,144]. Bone marrow MSC-EVs also attenuated OA progression in destabilization-induced murine models, with young donor-derived EVs demonstrating superior efficacy over aged counterparts [119,145]. Meanwhile, MSC-EVs derived from adipose tissue have shown chondroprotective effects [146]. However, IPFP-MSC-EVs from OA patients have exhibited pathogenic potential [57,99], underscoring the need to consider donor and disease-state dependencies of MSC-EV function. Various engineering approaches of MSC-EVs have improved therapeutic outcomes in OA, for example, EVs with charge-reversal modifications enhanced their cartilage penetration ability, miR-7704 overexpression in MSC-EVs amplified their effects on cartilage repair [147], and biomaterial-assisted delivery can prolong EV retention within the joint to enable sustained therapeutic effects [148]. In the field of stem cell and regenerative medicine, organoids constructed from reprogrammed stem cells can faithfully recapitulate the three-dimensional architecture and physiological microenvironment of native organs. The vesicles secreted by such reprogrammed stem cell derivatives are also inherently distinct from conventional EVs harvested from standard two-dimensional culture systems, making organoid technology a core research platform for disease modeling, mechanistic investigation and translational research. Derived from organoid systems, organoid tissue interstitial extracellular vesicles (OTEVs) represent a novel subtype of extracellular vesicles that bridges the research gap between organoid-derived EVs (OEVs) and tissue-derived EVs (TEVs). Compared with conventional EV models, OTEVs possess superior physiological fidelity and accurately recapitulate tissue microenvironmental signatures, offering innovative avenues for dissecting the pathological mechanisms of osteoarthritis, identifying novel diagnostic biomarkers, and developing microenvironment-tailored therapeutic strategies [149]. The gut–bone axis theory built upon intestinal organoid-derived EVs (IOEVs), which has been proposed in osteoporosis research, further corroborates this perspective. Organoid-derived EVs can act as physiologically relevant mediators of interorgan communication to regulate musculoskeletal remodeling, greatly broadening the application potential of organoid-originated EVs in mechanistic investigation and novel therapeutic development [150].

Despite encouraging results, several common limitations should be noted across preclinical studies evaluating MSC-EVs as therapeutic strategies for OA. Most current preclinical efficacy data on MSC-EVs are derived from mouse and rat OA models, where substantial interspecies differences exist in joint anatomy and pathophysiology compared to humans. For example, murine articular cartilage is considerably thinner (less than 50 μm versus approximately 2-4 mm in humans), lacks the distinct zonal organization seen in human cartilage, and resides in a joint with different biomechanical loading patterns, synovial volume, and immune cell composition. These differences can lead to overestimation of EV penetration depth and therapeutic efficacy when extrapolating from rodent models to the clinical setting. Furthermore, commonly used surgical OA models (e.g., destabilization of the medial meniscus, anterior cruciate ligament transection) induce acute, mechanically driven joint instability that may not fully recapitulate the progressive, metabolically influenced nature of human primary OA. While large animal models such as goats, sheep, horses, and dogs provide greater joint similarity to humans in anatomy, physiology, and biomechanics, they remain underutilized in EV research due to issues with ethics, accessibility, and cost. For clinical translation, EV efficacy should ideally be validated in at least one large animal model and be complemented by studies using primary human OA tissues where feasible. Throughout this review, we note the species context of cited studies where it influences the strength of translational conclusions. Another important limitation is that most preclinical studies administer MSC-EVs prophylactically or at early disease stages, while clinical OA typically presents at moderate-to-advanced stages, potentially leading to overestimation of therapeutic benefit. Cross-study comparisons are further complicated by substantial heterogeneity in EV sources (e.g., umbilical cord, bone marrow, adipose tissue), isolation methods, dosing regimens, and animal models [151,152]. Moreover, donor characteristics, cell passage number, and culture conditions significantly influence EV composition and bioactivity [145,151], while inconsistent reporting of EV characterization (including particle size, surface markers, cargo profiling, and dose metrics) limits reproducibility [153]. From a therapeutic perspective, low intra-articular retention due to rapid clearance and restricted diffusion through the dense cartilage matrix remains a major obstacle to achieving sustained bioavailability [66]. Finally, relatively few studies have systematically evaluated long-term safety, the immunogenicity of allogeneic MSC-EVs, or scalable manufacturing strategies required for clinical translation [154,155].

In summary, MSC-EVs have demonstrated promising mechanistic and preclinical evidence for modulating OA pathophysiology through anti-inflammatory, anti-senescence, and regenerative functions. However, standardization of production, enhanced targeting strategies, validation in late-stage disease models, and rigorous assessment of translational barriers are essential before clinical adoption. Future work should prioritize defining critical quality attributes, optimizing engineering approaches, and establishing robust potency assays to unlock the full therapeutic potential of MSC-EVs in OA.

### 5.2 Engineered EVs, biomaterial-assisted delivery and joint-targeted therapeutic platforms

Engineered EVs represent a biologically inspired extension of endogenous intercellular communication systems and are increasingly being developed as therapeutic delivery platforms for OA. Compared with synthetic nanocarriers, EVs possess intrinsic biological advantages, including complex membrane architecture, native cell-recognition properties, low immunogenicity, and efficient uptake by target cells [16,156]. These features are particularly relevant to OA, where therapeutic efficacy depends on simultaneously targeting multiple interconnected pathological processes, including inflammation, matrix degradation, and dysregulated tissue remodeling [157]. Despite their therapeutic promise, native EVs remain constrained by limited cargo loading capacity, rapid intra-articular clearance, poor retention and penetration within the dense cartilage matrix, donor-dependent heterogeneity, variable potency, and limited manufacturing scalability [109,142]. Therefore, current EV engineering strategies in OA are enhancing EV functionality through cargo loading, membrane modification, and tissue-specific targeting. Beyond direct injection, biomaterial-assisted delivery systems such as hydrogels, injectable matrices, and osteochondral scaffolds are emerging as important complementary platforms that improve EV retention, enable sustained release, and support spatially controlled delivery within the OA joint. More broadly, biomimetic therapeutic constructs integrating EVs within multiphasic scaffolds offer a strategy to simultaneously recapitulate native osteochondral architecture and deliver regenerative signals, advancing EV therapy from passive biologics toward programmable, tissue-integrated regenerative systems.

#### 5.2.1 Therapeutic cargo engineering and biological modulation of EVs in OA

Therapeutic engineering of EVs for OA is directed towards key pathological processes, including chondrocyte hypertrophy, apoptosis and senescence, ECM degradation, synovial inflammation, oxidative stress, macrophage polarization, and subchondral bone remodeling. Accordingly, EVs have been engineered to deliver regulatory nucleic acids, proteins, and small-molecule therapeutics that modulate disease-relevant signaling pathways. Among these, miRNA-enriched EVs have received considerable attention. EVs enriched with miR-7704 have been explored in OA mouse models for their therapeutic potential [147], and miR-223-bearing hUC-EVs have been shown to directly target NLRP3 mRNA to suppress inflammation and maintain chondrocyte homeostasis [59]. In parallel, EV-mediated delivery of anti-inflammatory or antioxidant compounds, including celecoxib and curcumin, has been used to reduce synovial inflammation, counteract oxidative stress, and rebalance anabolic-catabolic signaling within the OA joint [114,158].

Several EV loading strategies have been developed, each with distinct advantages and technical constraints. Electroporation is commonly used for loading nucleic acids and hydrophilic molecules [114], and genetic engineering of parent cells enables endogenous enrichment of specific cargos during EV biogenesis [159,160]. Compared with synthetic carriers such as liposomes and polymeric nanoparticles, EV-mediated delivery demonstrates enhanced cellular uptake and reduced cytotoxicity in preclinical models [14]. However, challenges remain in achieving reproducible loading efficiency, minimizing cargo aggregation or degradation during loading, and controlling release kinetics within the inflammatory OA microenvironment, where ROS, proteases, and altered pH may affect cargo stability [161]. Importantly, cargo engineering does not inherently overcome the low yield and manufacturing scalability of EV production, although emerging approaches such as 3D bioreactor culture systems or alternative biological EV sources (such as from photosynthetic microorganisms) offer innovative avenues to improve output and functional consistency [162].

A major engineering challenge is preserving the intrinsic biological functions of EV membranes while introducing exogenous cargo in a reproducible and controlled manner. Unlike chemically uniform synthetic nanocarriers, EVs vary substantially in lipid composition, membrane proteins, surface charge, stiffness, size distribution, and cargo profile depending on donor cell type, culture conditions, isolation methods, and disease context. These properties strongly influence loading efficiency, colloidal stability, biodistribution, cellular uptake, and immunomodulatory activity of EVs. Future OA-focused EV platforms will require rigorous physicochemical and functional characterization [163,164].

#### 5.2.2 Biomaterial-assisted delivery, surface functionalization and joint-targeted retention

Intra-articular injection remains the most widely used route for EV administration in OA, as it maximizes local exposure of joint tissues while minimizing systemic off-target distribution [165]. Nevertheless, direct injection alone does not guarantee sustained therapeutic bioavailability. After administration, EVs are rapidly diluted within synovial fluid and subject to clearance through lymphatic drainage, phagocytic uptake by synovial macrophages, enzymatic degradation, and mechanical loss during joint motion. These factors collectively shorten intra-articular residence time and often necessitate repeated dosing. Beyond EV retention, cartilage penetration presents an additional barrier. Articular cartilage is dense, avascular, and highly anionic due to its proteoglycan-rich ECM [166], which restricts nanoparticle diffusion based on size, charge, and surface chemistry. While smaller EVs may access superficial cartilage layers, homogeneous penetration into deeper zones remains limited. OA-associated cartilage matrix heterogeneity, with focal proteoglycan depletion, fissures, calcification, and altered hydration, further contribute to spatially inconsistent EV distribution.

To address these challenges, biomaterial-assisted delivery systems have emerged as a key strategy to enhance EV retention, protect cargo integrity, and enable controlled release in the OA joint. Hydrogels are particularly attractive because their high water content, tunable porosity, and injectable properties resemble aspects of the native ECM [167]. EV-loaded hydrogels can function as local depots for gradually releasing vesicles through diffusion, matrix degradation, mechanical loading, or disease-associated stimuli. Compared with bolus EV injection, hydrogel-based delivery may prolong intra-articular retention, reduce dosing frequency, and protect EVs from oxidative and enzymatic degradation [141,168].

Natural polymer hydrogels, including hyaluronic acid, collagen, gelatin, chitosan, alginate and fibrin, are widely investigated because of their biocompatibility and tissue-mimetic properties. Hyaluronic acid-based systems are especially relevant, given their established clinical use in viscosupplementation and ability to interact with CD44-expressing chondrocytes and synoviocytes [169,170]. Incorporating EVs into hyaluronic acid hydrogels may therefore combine lubrication, tissue retention, and biological signaling. Gelatin methacryloyl, collagen, and fibrin provide cell-adhesive motifs and matrix-like microenvironments, while chitosan and alginate offer tunable charge interactions and gelation properties, although these hydrogel materials require careful design to balance degradation rate, stiffness, and biocompatibility [171,172]. Synthetic and semi-synthetic hydrogels, such as polyethylene glycol-based networks, thermosensitive Pluronic systems, and supramolecular hydrogels, provide greater control over mechanical strength, mesh size, degradation kinetics, and release behavior [173]. These parameters critically determine EV release, which depends on hydrogel pore size, polymer concentration, crosslinking density, and EV-matrix interactions. A hydrogel network that is too loose may lead to rapid EV diffusion or burst release, whereas one that is too dense or adhesive may impair EV release and downstream cellular uptake. Thus, hydrogel design for EV delivery should balance local retention with therapeutic bioavailability.

Beyond hydrogels, microspheres, nanofibers, and scaffolds offer additional EV delivery options. Biodegradable polymeric microspheres can be used to encapsulate or adsorb EVs and enable sustained release as the matrix degrades [174]. Electrospun nanofibres and layered scaffolds can mimic ECM architecture and spatially localize EVs within joint tissues and interfaces, offering biomimetic platforms for integrated tissue repair. However, fabrication conditions such as organic solvents, high voltage, shear stress, lyophilization, and sterilization processes may compromise EV membrane integrity or cargo stability, requiring careful process optimization.

Surface engineering provides a complementary strategy to improve EV localization and cell specificity. Because EV biodistribution is strongly influenced by membrane proteins, lipid composition, surface charge, and adsorbed biomolecules, modifying the EV surface can alter tissue binding, matrix penetration, and cellular uptake [175]. Ligand-functionalized EVs displaying cartilage-binding peptides, collagen type II-binding motifs, chondrocyte-targeting ligands, or inflamed synovium-recognizing peptides may enhance retention in cartilage or synovial tissues and reduce nonspecific loss from the joint cavity [176]. Bone-targeting EV platforms have also gained attention due to the role of subchondral bone remodeling in OA pain and structural progression. EVs functionalized with bone-targeting motifs may improve delivery to mineralized tissues and modulate osteoclast-osteoblast coupling [177]. For example, DC-STAMP-decorated bacterial EVs have been engineered to deliver FRATtide selectively to pre-osteoclasts, thereby protecting the peptide from degradation and alleviating bone loss in an osteoporosis model, which may inform EV-based targeting of subchondral bone remodeling in OA [178]. Generally, mildly cationic modification of the EV surface may improve interactions with the negatively charged cartilage matrix, but excessive positive charge can increase cytotoxicity, aggregation, nonspecific protein adsorption, and phagocytic clearance [179]. Meanwhile, polyethylene glycol-based shielding can improve colloidal stability and reduce nonspecific uptake, but may also impair cellular internalization if overused [180].

Comparative studies indicate that EV delivery even with optimized platforms exhibit less predictable biodistribution than synthetic carriers, underscoring the need for rigorous *in vivo* validation [109,181]. It should be noted that no current targeting strategy achieves high-fidelity, cell-type-specific delivery simultaneously across cartilage, synovium, and subchondral bone as the major pathological compartments of OA. Future EV platforms will likely require modular or staged designs optimized to achieve effective compartment-specific therapy across the OA joint.

#### 5.2.3 Biomimetic regenerative platforms, translational challenges and future directions

Beyond functioning as passive delivery depots, advanced biomimetic scaffolds are increasingly designed to recreate instructive microenvironments for cartilage and osteochondral repair. In these systems, EVs act not simply as therapeutic cargos but as bioactive components of engineered matrices. EV-functionalized scaffolds can provide regenerative cues, regulate inflammation, recruit endogenous progenitor cells, and direct matrix deposition [182]. This is particularly relevant in OA, where persistently elevated inflammatory cytokines, ROS, matrix-degrading enzymes, and abnormal mechanical stress create a hostile environment for endogenous repair.

Rather than serving solely as structural implants for focal osteochondral defects, biomaterial-integrated EV platforms in OA may be designed to modulate pathological crosstalk across the osteochondral unit. This is particularly important because cartilage and subchondral bone exhibit distinct but interconnected degenerative changes during OA progression, including matrix breakdown, abnormal bone remodeling, vascular invasion, and altered mechanotransduction. In this context, spatially organized biomaterial systems such as layered hydrogels, injectable multiphasic matrices, or regionally functionalized scaffolds could enable compartment-specific EV delivery tailored to divergent microenvironments [[183], [184], [185]]. For example, cartilage-facing regions may preferentially release chondroprotective EVs to suppress catabolism and restore matrix homeostasis, whereas bone-facing compartments may deliver osteomodulatory EVs to regulate osteoclast-osteoblast coupling and subchondral sclerosis. Such spatial patterning may be valuable for treating OA as a whole-joint disease, helping to restore osteochondral homeostasis while interrupting pathogenic signaling between tissues. Stimuli-responsive biomaterials provide an additional layer of control by coupling EV release to disease-associated triggers such as reactive oxygen species, matrix metalloproteinases, acidic pH, or abnormal mechanical loading, thereby enabling more localized and temporally adaptive therapy [186]. These systems should be validated under physiologically relevant conditions, as buffer-based *in vitro* release assays may not accurately predict performance in the mechanically active and protease-rich joint cavity.

Despite encouraging preclinical findings, several materials-related and translational challenges remain. EV incorporation into biomaterials can alter vesicle conformation, diffusion, and cellular uptake. Strong electrostatic or covalent immobilization may improve EV retention but reduce internalization by target cells. Hydrogel degradation products, crosslinkers, or residual initiators may also affect chondrocyte viability or exacerbate synovial inflammation. Mechanical compatibility is equally important, as materials that are too soft may be rapidly displaced, whereas overly stiff materials may impair lubrication or provoke mechanical irritation. In addition, sterilization, storage, lyophilization, and transport must preserve both biomaterial integrity and EV bioactivity.

Dosing and pharmacokinetics also remain unresolved issues [187]. Across preclinical OA studies, EV dosage is variably reported as particle number, protein concentration, or preparation volume, making cross-study comparisons difficult. Particle number alone may not reflect biologically active cargo, whereas protein concentration may be confounded by non-vesicular contaminants. Hence, none of these metrics reliably reflects EV functional potency, which is influenced by donor cell source, culture conditions, isolation method, and manufacturing batch [188]. Heterogeneity in EV size, surface markers, and donor-cell origin further complicates reproducible targeting [151]. Quantitative pharmacokinetic-pharmacodynamic studies linking EV dose, intra-articular retention, tissue penetration, and therapeutic response remain scarce. Following injection, EVs are rapidly diluted in synovial fluid and cleared from the joint cavity through lymphatic drainage and phagocytic uptake by synovial macrophages, with rapid intra-articular clearance observed in small-animal models, where local retention only lasts several hours to a few days [165,189]. Repeated injection regimens, while potentially compensating for rapid EV clearance, raise concerns about cumulative joint trauma, infection risk, and patient compliance. Real-time imaging strategies to monitor EV biodistribution and clearance, particularly in large animal models, will be critical for defining therapeutic windows and optimizing dosing frequency.

Scalable production also remains a major bottleneck. Conventional 2D cell culture yields insufficient EV quantities for clinical translation, whereas large-scale bioreactors, 3D culture systems, and cell-derived nanovesicle techonologies may improve output but can alter EV composition and potency [190]. Standardized potency assays, reproducible manufacturing workflows, and regulatory-grade quality control frameworks are urgently needed. Future preclinical studies should incorporate more quantitative endpoints, including biodistribution, retention, tissue-specific uptake, and comparative efficacy of EVs against modified systems such as hydrogel-encapsulated EVs, ligand-modified EVs, and synthetic nanoparticles. Repeated-dose safety, immunogenicity, ectopic tissue effects, pro-fibrotic responses, and interactions with existing OA treatments also require systematic evaluation.

Ultimately, the future of EV engineering in OA will likely lie in integrated therapeutic platforms that combine rational cargo design, biomaterial-assisted retention, tissue-targeting surface engineering, and disease-responsive release. Achieving this will require a deeper mechanistic understanding of EV-material interactions, standardized dosing and manufacturing frameworks, and rigorous validation in clinically relevant models.

### 5.3 Comparison with cell-based therapies

MSC transplantation has been extensively investigated as a regenerative strategy for OA, leveraging their paracrine signaling to modulate inflammation, promote chondroprotection, and stimulate tissue repair [191,192]. The emergence of EV-based therapies stems from the recognition that many of these therapeutic effects are mediated by secreted vesicles carrying bioactive cargo that regulate chondrocyte senescence, ECM homeostasis, macrophage polarization, and synovial inflammation [18,19,109]. However, while EVs function as critical effectors of MSC activity, they should not be regarded simply as “cell-free surrogates” as their biological behaviour, pharmacology, and translational profiles differ substantially from live-cell therapies.

MSC therapies exert multimodal effects through dynamic secretion of trophic factors, immunomodulatory interactions, and adaptive responses to the local microenvironment, with limited but potentially relevant engraftment in periarticular tissues [191,193]. In contrast, EVs deliver predefined molecular cargoes determined by the donor-cell state and manufacturing conditions. Therapeutic EV cargos, such as miR-223-3p [194] and miR-146b-5p [152], have been shown to suppress pro-inflammatory pathways such as NF-κB and TRAF6, enhance autophagy, and promote macrophage polarization towards a reparative phenotype [195]. However, EV efficacy remains highly context-dependent, influenced by donor source (e.g., umbilical cord, adipose tissue, bone marrow), preconditioning strategies (e.g., LPS, cytokines, electric or magnetic fields), and engineering modifications [100,104,196]. Unlike living MSCs, EVs cannot dynamically respond to evolving disease microenvironments, which may limit durability in chronic or metabolically driven OA phenotypes [80,197].

From a safety perspective, MSC therapies carry theoretical risks of immune incompatibility (particularly from allogeneic sources), ectopic differentiation, and tumorigenicity, though clinical evidence for these remains limited [5,198,199]. EVs generally exhibit lower immunogenicity and negligible tumorigenic risk due to their acellular nature and inability to proliferate [199]. Nevertheless, EV safety is not absolute as batch-dependent contaminants (e.g., from culture medium), off-target biodistribution, and pathogenic cargo from senescent or diseased donor cells (e.g., OA joint-derived MSC EVs) may introduce biological risks [57,200]. Additionally, unmodified EVs face steric and electrostatic barriers within dense cartilage ECM, reducing intra-articular retention and bioavailability.

Both modalities suffer from manufacturing and standardization challenges. MSC products are influenced by donor age, passage number, and culture conditions [145,151]. requiring stringent GMP-compliant expansion, viability monitoring, and cryopreservation workflows. EV production faces additional complexity due to low native yield, necessitating bioprocess optimization, preconditioning, or engineering to improve scalability and potency [155,162]. Standardizing EV isolation methods (e.g., ultracentrifugation versus tangential-flow filtration), resolving heterogeneous subpopulations (e.g., exosomes versus ectosomes), and establishing biologically relevant potency assays tied to functional cargo remain major bottlenecks [147,200]. Regulatory classification also remains ambiguous for EVs, as they may be categorized as drugs, biologics, or advanced therapy medicinal products (ATMPs), complicating global harmonization [133].

Rather than framing EVs as inherently superior to MSCs, current evidence supports a complementary therapeutic paradigm. EVs address several practical limitations of cell therapy, including reduced safety concerns, simplified storage and transport, and greater feasibility for repeat dosing [199]. Conversely, MSCs retain advantages in dynamic microenvironment sensing and sustained paracrine signaling, which may be beneficial in complex, progressive joint pathophysiology [197]. Emerging combinational strategies, including EV-primed MSCs and EV-hydrogel composites, may yield synergistic effects. Ultimately, therapeutic selection will likely depend on disease biomarker, pathological phenotype, target joint compartment, and clinical practicality. Advancing both platforms will require mechanistic clarity, standardized manufacturing and reporting, and rigorous comparative studies to establish reproducible clinical benefit [9,11,133].

## 6 Challenges and limitations in current EV research

EV research has catalyzed transformative conceptual advances in understanding OA pathophysiology, revealing complex intercellular communication networks among chondrocytes, synoviocytes, macrophages, subchondral bone cells, and MSCs. Across this review, EVs emerge as central mediators of disease propagation, tissue crosstalk, and repair, while also offering opportunities for biomarker development and therapeutic intervention [201]. However, the field continues to face methodological, biological, and translational limitations that hinder robust mechanistic interpretation, reproducibility, and clinical implementation. Many of these challenges are not unique to OA but reflect the broader immaturity of EV science as a field. Importantly, they should be viewed as areas for refinement rather than barriers to progress.

A major limitation remains the lack of standardized methodologies for EV isolation, characterization, and cargo analysis. Substantial variability exists across isolation platforms, including ultracentrifugation, size-exclusion chromatography, microfluidic approaches, and immunoaffinity capture, each producing distinct EV yields, purities, and subtype enrichment profiles [202,203]. Likewise, analytical techniques such as nanoparticle tracking analysis, cryo-electron microscopy, and flow cytometry differ in sensitivity, resolution, and reporting outputs, contributing to inconsistent reporting of EV size, concentration, and surface markers [202]. This methodological heterogeneity complicates cross-study comparisons, weakens biomarker validation in synovial fluid and plasma [117,204], and limits therapeutic reproducibility. While community-driven initiatives such as MISEV recommendations have established important reporting standards, implementation remains inconsistent particularly in complex biological matrices relevant to OA.

EV heterogeneity presents equally significant biological and technical challenges. Within the joint, EVs are released by diverse cell populations, including chondrocytes, FLSs, bone cells (osteocytes, osteoblasts, osteoblasts), and immune cells, each generating distinct subpopulations with context-dependent molecular cargo and biological functions [1,4]. Bulk EV analyses often obscure these functionally important subsets. Moreover, distinguishing pathogenic from reparative EV populations, and assigning their contributions to inflammation, ECM degradation, senescence, or aberrant bone remodeling remains technically challenging [3]. Current technologies still have limited capacity to resolve EV origin, biodistribution, cargo dynamics, and intercellular trafficking *in vivo* with sufficient precision, constraining mechanistic understanding.

Reproducibility and scalability represent further barriers to translation. Batch-to-batch variability in EV production, shaped by donor age, passage number, culture conditions, and priming strategies, substantially affects experimental consistency [145,151]. Conventional cell culture systems also yield low EV quantities, limiting preclinical scalability and increasing manufacturing costs [142,162]. In addition, contamination from serum-derived vesicles, protein aggregates, or non-vesicular components remain a persistent challenge for product purity and quality control. While engineering strategies such as 3D bioreactor systems, hydrogel-assisted delivery, and EV cargo or surface functionalization to improve EV yield, cargo loading, targeting, or retention have offered promising solutions, they also introduce additional layers of complexity in manufacturing and quality control. Without standardized potency assays linking EV composition to measurable biological function [162], therapeutic reproducibility across laboratories remains difficult to achieve.

The translational and regulatory landscape for EV-based therapies remains underdeveloped. EV-based therapeutics occupy an ambiguous regulatory space, with ongoing uncertainty regarding whether they should be classified as biologics, drugs, or ATMPs. Evolving frameworks lack consensus on critical quality attributes, stability requirements, or long-term safety profiles [156]. Concerns regarding immunogenicity, off-target effects, or unintended modulation of disease pathways (e.g., pro-inflammatory EVs exacerbating OA) necessitate rigorous *in vivo* safety studies [141,154]. Furthermore, the absence of validated potency assays complicates dose standardization and batch release criteria. Although engineered EVs and biomaterial-integrated delivery systems such as hydrogels and scaffolds aim to improve retention and efficacy, their clinical feasibility depends on scalable GMP-compliant manufacturing workflows and robust regulatory frameworks.

These limitations have direct implications for OA-specific applications. In biomarker development, inconsistent EV isolation and characterization methods undermine the reliability of plasma or synovial fluid EV signatures for early OA detection, prognostic stratification, and treatment monitoring [117,204]. In therapeutics, issues with biological heterogeneity and scalability delay the transition of promising preclinical findings into clinical trials [59]. Addressing these challenges will require coordinated advances across multiple disciplines, including improved single-EV analytics, standardized biobanking, OA-relevant potency assays, and more predictive preclinical models. Integrating multi-omics profiling, computational modeling, and quantitative *in vivo* tracking may further strengthen mechanistic insight and therapeutic optimization [125]. By framing current constraints as catalysts for innovation, the field can accelerate the maturation of EV science toward clinically actionable platforms in OA.

## 7 Future perspectives

The field of EVs research in OA is transitioning from descriptive cataloguing of EV sources and cargoes towards mechanistic dissection of joint-wide communication networks. This shift reflects a broader conceptual evolution, positioning EVs not only as biomarkers or therapeutic carriers but as active regulators of disease progression and repair. Key priorities include resolving EV heterogeneity, improving scalable manufacturing, and developing robust biomarker and potency frameworks. Below, we outline critical future directions to advance EV science from biology towards clinical application in OA.

### 7.1 Emerging technologies in EV research

Technological innovation will be central to resolving longstanding challenges in EV heterogeneity, isolation fidelity, and functional validation. Advances in high-resolution flow cytometry now enable more precise profiling of EV subsets through combined surface marker and cytokine analysis [53,205], revealing size-dependent associations with radiographic OA progression [56]. Novel imaging modalities, such as *in situ* cargo detection without prior EV isolation, and engineered tracking systems (e.g., macrophage-derived EVs for subchondral bone targeting) enhance the spatial resolution of EV trafficking within joint compartments [14]. However, technological sophistication alone is insufficient, as the value of advanced technologies lies in defining which EV populations mediate specific signals, under which pathological conditions, and across which tissue interfaces.

### 7.2 Integration of multi-omics approaches

**Fig. 6:**
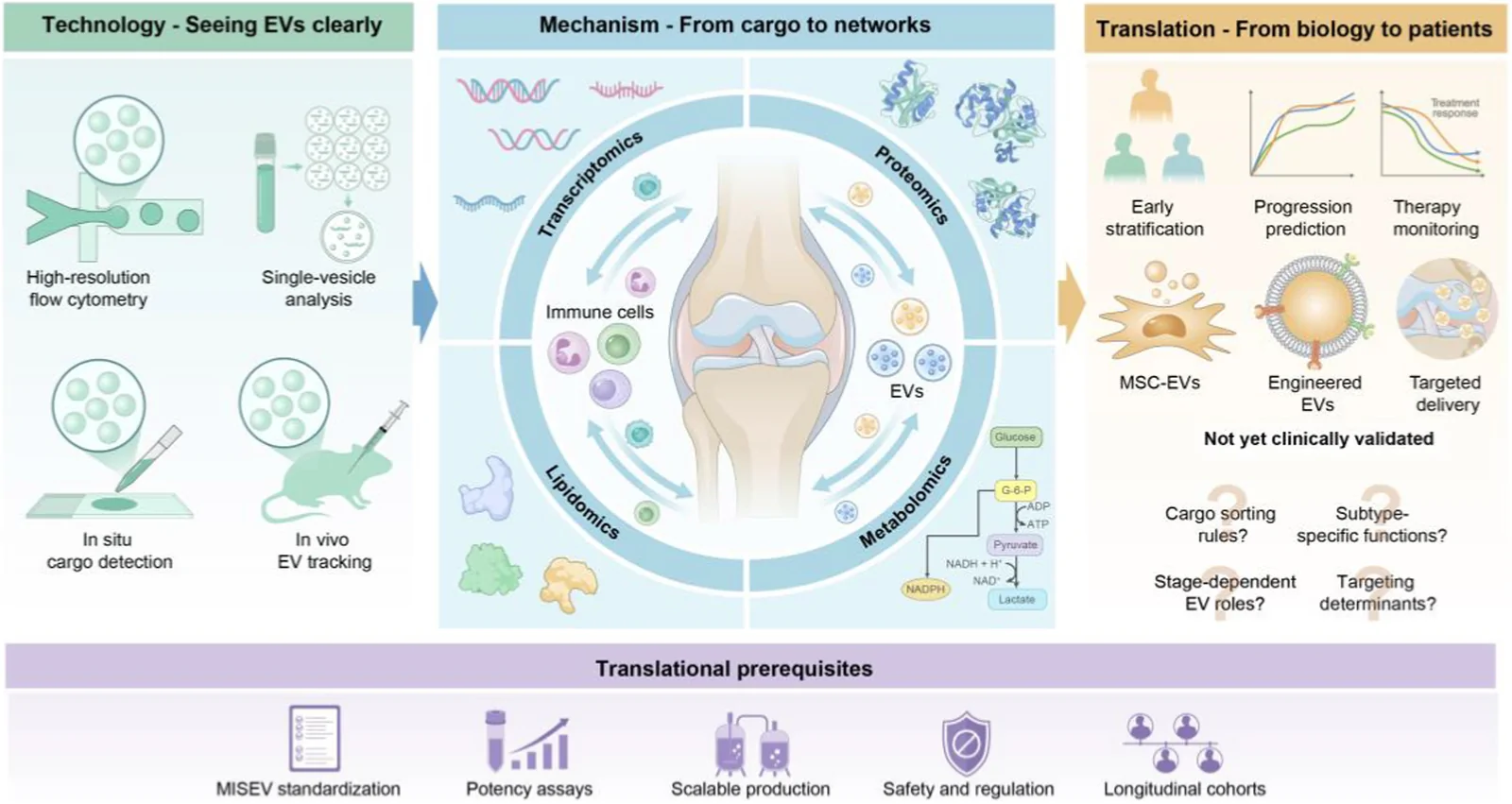
From EVs resolution to clinical translation: integrating technology, mechanism and therapeutic development in OA.

Deciphering EV-mediated communication networks in OA necessitates systems-level integration of transcriptomic, proteomic, lipidomic, and metabolomic datasets (Fig. 6). Beyond individual miRNAs, diverse cargo classes such as circulating RNAs (e.g., miR-23b-3p from osteocyte-derived EVs) [15], including those enriched in exosomes [206,207], and surface lipid signatures [208] collectively shape recipient cell responses in cartilage, synovium, and subchondral bone. Multi-omics integration can decode context-dependent EV functions, for instance, SASP-EVs have been found to drive inflammation in early OA [209,210]. Databases such as EVmiRNA2.0 are increasingly enabling cross-study cargo comparisons, although harmonization of omics workflows and metadata remains essential to distinguish EV signatures from technical artifacts. The ultimate goal is not simply to catalogue EV cargo complexity, but to reconstruct functional communication networks that explain disease-stage progression, tissue crosstalk, and OA phenotypes.

### 7.3 Clinical translation and personalized medicine

EVs hold considerable potential for improving OA management through enabling biomarker discovery and cell-free therapeutics (Fig. 6). Therapeutically, engineered EVs exemplify rational design strategies, such as curcumin-loaded EVs to enhance bioavailability [211]. However, translation into clinical practice will require adherence to standardized protocols for EV production and more rigorous validation, particularly considering variable factors such as donor age [151] and inconsistent yields that limit scalability. Longitudinal clinical studies will be essential to define how EV profiles evolve relative to OA symptommatic progression, imaging findings, and treatment response. In the longer term, personalized EV-based approaches may emerge, where EV source, cargo composition, or targeting strategies are matched to patient-specific disease biology.

### 7.4 Open questions and research directions

Fundamental questions remain in our understanding of EV biology in the context of OA (Fig. 6). Key gaps include understanding how specific cargos are selectively sorted into distinct EV subtypes (e.g., migrasomes versus exosomes) during joint stress, as well as how lipid composition governs EV targeting to chondrocytes versus macrophages [208]. The temporal dynamics of EV signaling in OA are also poorly understood, including whether pathogenic EVs derived from FLSs or IPFP-MSCs predominate during early OA, and whether reparative EV populations prevail at later stages [4]. The dual and context-dependent roles of EVs, capable of either amplifying degeneration (e.g., OA chondrocyte-derived EVs) or supporting repair (e.g., MSC-EVs), highlight the need for cell-of-origin-resolved and tissue-specific analyses rather than bulk EV profiling. Furthermore, emerging concepts such as interspecies EV communication, including plant-derived phytosomes, and cross-kingdom RNA trafficking [212] warrant cautious, mechanism-focused exploration as potential avenues for innovative EV delivery strategies. Addressing these uncertainties will be critical for moving from associative observations to predictive and intervention-ready EV biology.

### 7.5 Integrative closing perspective

EVs represent a unifying framework for decoding multicellular dialogue across the OA joint, from the synovium to cartilage and subchondral bone (Fig. 6). They bridge molecular dysregulation (e.g., TGF-β signaling [14], SASP [15]) and clinical heterogeneity, offering a lens to reframe OA as a network disorder rather than isolated tissue failure. Realizing this potential will require three parallel commitments: clearer biological definitions of EV subtypes and functions, stronger methodological consistency across studies, and realistic translational strategies that address manufacturing, safety, and regulatory constraints. Progress will depend on interdisciplinary collaboration spanning musculoskeletal biology, bioengineering, computational modelling, and clinical science. While EV-based diagnostics and therapies remain at an early stage, continued integration of mechanistic biology with engineering and clinical research may ultimately establish EVs as precision tools for monitoring, stratifying, and modifying OA progression.

Advancing EV biology towards clinical impact in OA requires coordinated progress across three interconnected domains: technology, mechanistic understanding, and translational implementation. Technology: increasing resolution of EV characterization highlights emerging approaches that clarify EV heterogeneity beyond bulk populations, including high-resolution flow cytometry, single-vesicle analysis, *in situ* cargo detection, and *in vivo* EV tracking, enabling more precise understanding of EV subtypes, cellular origin and biodistribution. Mechanism: from EV cargo to communication networks, mechanistic advances emphasize that EV function in OA is context-dependent and shaped by multimodal cargo landscapes across transcriptomic, proteomic, lipidomic, and metabolomic layers. Through these cargo-driven signaling networks, EVs coordinate whole-joint communication linking cartilage, synovium, subchondral bone, and immune compartments, thereby contributing to both pathogenic and reparative cellular programs. Translation: from EV biology to clinical application, the emerging potential of EV-informed OA management strategies includes early disease stratification, progression prediction, and therapy monitoring, alongside the development of MSC-derived and engineered EVs for targeted intra-articular delivery. However, EV-based diagnostics and therapeutics remain insufficiently clinically validated, and successful translation will require rigorous standardization (e.g., according to MISEV recommendations), robust potency assays, scalable manufacturing, safety and regulatory frameworks, and longitudinal patient cohorts. Together, this framework underscores the need for hypothesis-driven integration across discovery technologies, mechanistic network biology, and therapeutic engineering to unlock EV-based disease-modifying interventions in OA.

## 8 Conclusions

EVs constitute a fundamental component of intercellular communication within the OA joint, integrating molecular signals across cartilage, synovium, subchondral bone, and immune compartments. By transferring regulatory RNAs, proteins, lipids, and stress-associated molecules, EVs actively shape inflammatory signaling, matrix remodeling, and cell fate decisions in a context-dependent manner, supporting a systems-level view of OA as a disorder of disrupted multicellular communication rather than isolated tissue failure. EVs hold significant promise as OA biomarkers and therapeutic platforms, particularly through innovative advances such as characterization technologies, engineered EVs, and biomaterials-assisted delivery platforms. However, their clinical translation remains limited by vesicle heterogeneity, incomplete functional annotation, and insufficient methodological standardization. Future progress will depend on rigorous mechanistic interrogation, integrative multi-omics approaches, and translational pragmatism, positioning EV biology not as an immediately deployable clinical solution, but as a powerful conceptual and therapeutic framework for refining OA stratification, monitoring disease evolution, and guiding targeted intervention.

## 9 Literature search and scope

This narrative review draws on a systematic search of the PubMed, Web of Science, and Scopus databases for English-language articles published between January 2015 and May 2026. Search terms included "extracellular vesicles" OR "exosomes" OR "microvesicles" OR "small EVs" combined with "osteoarthritis" OR "OA" OR "joint degeneration" OR "cartilage" OR "synovium" OR "subchondral bone". Additional targeted searches incorporated terms such as "MSC-EVs," "engineered EVs," "biomaterial," "hydrogel," "biomarker," and "intercellular communication." Reference lists of key articles and recent reviews were manually screened to identify additional relevant studies. Studies were selected based on their mechanistic depth, methodological rigor, and relevance to OA joint tissue crosstalk and translational applications, with priority given to publications in the last five years. Preclinical studies (*in vitro* and *in vivo* models), human observational studies, and clinical trials were all considered; the nature and level of evidence are noted throughout the manuscript where major inferences are drawn. The study scope encompasses EV biology in OA-relevant joint tissues, diagnostic and prognostic biomarker development, and therapeutic strategies including MSC-derived EVs, engineered EVs, and biomaterial-assisted delivery platforms. This review does not employ formal meta-analysis or systematic review methodology, and the selection of representative studies reflects the authors’ synthesis of the most informative and rigorously conducted work available in the current literature.

## CRediT authorship contribution statement

**Zhen Yang:** Writing – original draft, Formal analysis, Data curation, Conceptualization. **Qiyuan Lin:** Formal analysis, Conceptualization. **Hao Li:** Software, Methodology. **Senyang Xiao:** Software. **Hebin Ma:** Software, Methodology. **Jianhao Lin:** Supervision. **Dan Xing:** Writing – review & editing, Supervision.

## Ethics approval and consent to participate

Not applicable. This article is a review and does not contain any studies with human participants or animals performed by any of the authors.

## Declaration of competing interest

The authors declare no conflict of interest.
