# Supplementary material for: From pathogenic to reparative: Context-Dependent function of extracellular vesicles in osteoarthritis
Source: Bioact Mater. 2026 Sep 8;68:397–417. doi: 10.1016/j.bioactmat.2026.09.005 (PMC13573681; doi:10.1016/j.bioactmat.2026.09.005)
Supplement: Supplementary file 1 [file mmc1.docx]

**Supplementary Tables**

Extracellular Vesicles in Osteoarthritis: From Pathogenic to Reparative

Table 1. Summary of EV sources, cargo, target cells, functional effects, and OA-related mechanisms.

| **EV Source** | **Key Cargo** | **Target Cells** | **Functional Effects** | **OA-Related Mechanisms** |
| --- | --- | --- | --- | --- |
| **PATHOGENIC EVs (Pro-OA)** | | | | |
| **OA Chondrocytes (senescent / inflammatory)** | SASP factors (IL-1β) [66,70,103]; Cx43 [103]; miR-155 [55]; MMP-13, ADAMTS-5 [78]; senescence-inducing miRNAs [70]; NLRP3 inflammasome-activating cargo (drives IL-1β secretion in macrophages) [87] | Neighbouring chondrocytes, synovial fibroblasts, macrophages, subchondral bone cells | Propagate cellular senescence [66,70,103]; amplify catabolic enzyme secretion (MMP-13, ADAMTS-5) and suppress Col II/aggrecan [78]; activate macrophage NLRP3 inflammasome → mature IL-1β secretion [87]; initiate pathological cartilage calcification via mineral-containing autophagosomes [65] | Cx43-mediated SASP transfer drives paracrine senescence across joint tissues [103]; OA chondrocyte EVs establish a self-perpetuating cartilage–synovium inflammatory cycle via NLRP3/IL-1β [87]; senescent chondrocyte EVs deliver SASP components and senescence-inducing miRNAs, amplifying joint-wide tissue degeneration [66,70,103] |
| **OA Fibroblast-Like Synoviocytes (FLS)** | miR-19b-3p (induces GPX4 downregulation → ferroptosis) [85]; miR-146a (downregulated in OA-FLS; overexpression reverses pathology via M1→M2 repolarization) [86]; altered miRNA signatures and pro-inflammatory mediators [4,67,96] | Chondrocytes, macrophages | miR-19b-3p induces chondrocyte ferroptosis (GPX4↓) and apoptosis while sustaining M1 macrophage polarization [85]; miR-146a downregulation promotes inflammatory cascades; overexpression reverses effects via M1-to-M2 repolarization [86]; EV secretion increases with synovial inflammation, establishing feed-forward loop of joint degeneration [4,67,96] | IL-1β-stimulated FLS secrete miR-19b-3p-enriched EVs that drive chondrocyte ferroptosis via GPX4 suppression [85]; endogenous miR-146a acts as a protective brake—its loss in OA-FLS-EVs amplifies inflammatory signalling, while restoring miR-146a promotes M2 macrophage repolarization [86]; FLS→macrophage→chondrocyte feed-forward inflammatory axis [4,67] |
| **M1 Macrophages (pro-inflammatory)** | TNF-α, IL-6 [61,88]; pro-inflammatory cytokines [68]; FMRP-selectively sorted cargo [68] | Chondrocytes, macrophages | Induce pro-inflammatory response in chondrocytes (IL-1β, TNF-α secretion) [68]; accelerate cartilage degradation in murine models [68]; exacerbate chondrocyte apoptosis and matrix degradation [95]; sustain M1 phenotype and synovial inflammation [61,88] | M1 macrophages drive synovial inflammation through antigen presentation and cytokine signalling (TNF-α, IL-6) [61,88]; FMRP-mediated selective cargo sorting in inflammatory macrophage EVs [68]; M1/M2 imbalance constitutes a central pathogenic mechanism in OA chronic low-grade inflammation [88] |
| **Osteocytes (pathological mechanical loading)** | miR-23b-3p [15,78,110,113]; miR-210-3p (reduced in OA) [113] | Chondrocytes, endothelial cells at osteochondral interface | miR-23b-3p targets OTUD4 in chondrocytes → impairs mitophagy → elevates MMP-13 and ADAMTS-5, reduces Col II and aggrecan [15,78,113]; decreased miR-210-3p associated with excessive vascular invasion at bone–cartilage interface [113] | Osteochondral crosstalk disruption: bone-derived EVs directly modulate chondrocyte catabolic function [15,113]; miR-23b-3p/OTUD4/mitophagy axis as a key driver of the chondrocyte catabolic shift [15,78,110]; pathological mechanical loading reprograms osteocyte EV cargo [15,110] |
| **Pre-osteoclasts (metabolic OA)** | COX2-PGE2 axis factors [110,124]; senescence-associated secretome [124] | Osteoblast progenitors, osteoclasts | Stimulate OB progenitor differentiation [110,124]; suppress OC maturation [110,124]; drive pathological subchondral bone plate thickening prior to cartilage damage [110] | Senescent pre-osteoclast secretome activates COX2-PGE2 axis → uncoupled bone remodelling [110,124]; metabolic syndrome-associated OA drives pre-osteoclast senescence [124]; EV-mediated OB/OC uncoupling precedes overt cartilage degeneration [110] |
| **Senescent BM-MSCs (subchondral bone)** | SASP factors [110,125,126]; PGE2 [110,127] | Osteoblasts, osteoclasts, chondrocytes | Contribute to subchondral bone sclerosis [110,125,126]; impair microenvironmental homeostasis [125,126]; PGE2 drives sensory nerve ingrowth and structural deterioration via EP4 receptor [110,127] | SASP-EV-mediated paracrine senescence propagation within subchondral bone niche [125,126]; PGE2/EP4 axis modifiable by COX2 inhibition or targeted EV delivery [110,127]; disrupted OB-OC coupling through altered EV signalling [110] |
| **OA Infrapatellar Fat Pad (IPFP)** | Pro-inflammatory mediators [75,97]; mtDNA [57] | Chondrocytes | Propagate inflammation and ECM degradation [75,97]; GW4869 inhibition of EV release attenuates inflammatory cascades in chondrocytes [97]; mtDNA transfer may contribute to pathogenic effects [57] | IPFP-derived sEVs from OA patients propagate chondrocyte inflammation [75,97]; IPFP-MSC-EVs may accelerate OA progression via mtDNA transfer and pathogenic EV subpopulations [57]; local adipose tissue–EV-mediated link between metabolic dysfunction and joint inflammation [75] |
| **Hybrid EVs (hEVs) (OA synovial fluid)** | Co-express pro-inflammatory macrophage and chondrocyte surface markers [51,56]; TOMM20 (mitochondrial marker) [51] | Chondrocytes | Induce chondrocyte mitochondrial damage [51,56]; hEV abundance correlates with OA clinical severity [56] | Single-vesicle analysis identifies hEVs as a novel subpopulation reflecting pathological macrophage–chondrocyte interactions [51,56]; TOMM20+ EV subsets elevated in OA vs trauma controls, suggesting mitochondrial stress signatures as disease-stage biomarkers [51,92] |
| **PROTECTIVE / HOMEOSTATIC EVs (Anti-OA)** | | | | |
| **Healthy FLS (homeostatic)** | Annexin A1 (ANXA1) via PPARγ/ESCRT axis [84] | Chondrocytes | ANXA1 inhibits ERK phosphorylation and activates autophagy in chondrocytes, preserving cartilage homeostasis [84] | PPARγ controls ESCRT-dependent EV biogenesis in FLS; ANXA1 delivered via EVs maintains chondrocyte health by suppressing ERK signalling [84]; contrasted with OA-FLS EVs that drive ferroptosis and inflammation [85,86] |
| **M2 Macrophages (anti-inflammatory)** | IL-10 [62,95,99]; anti-inflammatory mediators | Chondrocytes, M1 macrophages | Promote tissue repair and inflammation resolution [62,95]; safeguard chondrocyte integrity [62]; MSC-EVs reprogram M1→M2, restoring immune microenvironment homeostasis [67,95] | M2 macrophage EVs deliver IL-10 to protect chondrocytes [62]; M1/M2 imbalance is a central OA pathogenic mechanism [88]; UC-MSC-EVs drive M2-like polarization via IL-10 secretion [59,95,111] |
| **Neutrophils** | SFRP5 (secreted frizzled-related protein 5) [75,101]; anti-inflammatory mediators [101] | Chondrocytes | Promote proteoglycan synthesis [75,101]; reduce catabolic enzyme expression [75,101]; contribute to chondroprotection and attenuation of synovial inflammation [75,101] | Upregulation of SFRP5 in chondrocytes via neutrophil-derived EVs [75,101]; modulation of Wnt signalling toward anabolic outcomes [101] |
| **Platelet-Rich Plasma (PRP)** | Growth factors [95,113]; anti-inflammatory cargo [95,113] | Synovial macrophages, chondrocytes | Suppress pro-inflammatory macrophage activation [95,113]; promote reparative macrophage functions [95,113]; reduce synovitis and protect cartilage in preclinical models [95,113] | Modulation of macrophage functional plasticity toward reparative phenotypes [95,113]; PRP-EVs traverse tissue barriers to deliver immunomodulatory signals into synovial fluid [99] |
| **MSC-DERIVED THERAPEUTIC EVs** | | | | |
| **UC-MSC-EVs (Umbilical Cord)** | miR-223 (targets NLRP3 mRNA, suppresses pyroptosis) [59,141]; anti-inflammatory miRNAs and proteins [59,98,99]; METTL3-interacting factors (reduce m6A of NLRP3 in macrophages) [2] | Chondrocytes, synovial macrophages, synoviocytes | Reduce cartilage degradation and chondrocyte apoptosis [59,142]; suppress synovial inflammation [59,142]; promote ECM anabolism and Col II synthesis [59]; drive M2 macrophage polarization and increase IL-10 secretion [59,111]; enhance autophagy [59]; reduce NLRP3 inflammasome activation [2,59] | miR-223 directly targets NLRP3 mRNA to suppress inflammation and maintain chondrocyte homeostasis [59,141]; METTL3-mediated m6A modification of NLRP3 in macrophages [2]; multi-target chondroprotection combining anti-inflammatory, anti-apoptotic, and pro-anabolic effects [59,111,142] |
| **BM-MSC-EVs (Bone Marrow)** | miR-146b-5p (targets TRAF6) [148,163]; miR-223-3p [163,192]; growth factors, immunomodulatory proteins [117,143] | Chondrocytes, macrophages, osteoblasts/osteoclasts | Attenuate OA progression [117,143]; suppress NF-κB and TRAF6 pathways [148,163]; reduce chondrocyte apoptosis and senescence [117]; promote M2 macrophage polarization [117,163]; attenuate subchondral bone deterioration [117]; young donor-derived EVs demonstrate superior efficacy over aged donors [117,143] | miR-146b-5p suppresses TRAF6/NF-κB pro-inflammatory signalling [148,163]; miR-223-3p promotes macrophage polarization toward reparative phenotype [163,192]; donor-age-dependent therapeutic efficacy highlighting importance of cell source selection [117,143] |
| **AD-MSC-EVs (Adipose Tissue)** | Anti-oxidative and anti-inflammatory cargo [100]; miR-126-3p (upregulated upon curcumin priming) [156] | Chondrocytes | Mitigate oxidative stress by modulating NOX4-related pathways and reducing ROS-driven inflammation [75,82,100]; curcumin-primed EVs reverse IL-1β-induced catabolic responses via miR-126-3p upregulation [156] | NOX4/ROS pathway modulation linking oxidative stress suppression to reduced inflammation and ECM preservation [75,82,100]; curcumin priming enhances anti-catabolic miRNA cargo for chondroprotection [156] |
| **Preconditioned MSC-EVs (hypoxia / cytokine / 3D culture)** | 2D monolayer: miR-27a-3p, TGF-β/STAT3/BCL-2, Raf/MEK/ERK, Wnt/β-catenin pathways [62,89-91]; Hypoxia (1–3% O₂): VEGF pathway activation, 2–3× EV yield [62,78,92,93]; 3D scaffold: integrin-mediated ECM proteins [62,89,94]; LPS priming: NF-κB-driven miR-483-5p and miR-140-3p enrichment [62,93,95] | Chondrocytes, macrophages, endothelial cells | 2D monolayer: basal anti-inflammatory function via M2 polarization/IL-10 [62]; Hypoxia: enhanced pro-angiogenic capacity and EV yield [62,78]; 3D scaffold: ECM-enriched EVs with improved functional stability [62,89]; LPS priming: enhanced immunomodulatory potency via NF-κB-driven cargo remodelling [62,93,95] | Culture conditions govern EV identity and therapeutic function—identical MSCs yield functionally divergent EVs depending on conditioning [62]; HIF-1α and VEGF pathways under hypoxia [62,78]; integrin-mediated mechanotransduction in 3D culture reshapes EV cargo toward ECM-regenerative phenotypes [62,89]; inflammatory priming exerts biphasic effects [62,93,95] |
| **ENGINEERED / MODIFIED EVs (Rational Design)** | | | | |
| **miRNA-Enriched / Genetically Engineered EVs** | miR-27b-3p (targets CSF-1 axis) [96]; miR-7704 [132,141,145]; miR-223 (targets NLRP3) [59,141]; circHIPK3/miR-124-3p/MYH9 axis [19]; circCREBBP knockdown (promotes TGF-β2/ECM anabolism) [81]; SNHG5 (reprograms autophagy and macrophage polarization) [111] | Chondrocytes, synovial macrophages | miR-27b-3p-enriched sEVs selectively reprogram macrophages via CSF-1 targeting [96]; miR-7704 overexpression amplifies cartilage repair and reduces MMP-13 [132,141,145]; miR-223-bearing EVs suppress NLRP3 inflammasome [59,141]; circHIPK3 regulates miR-124-3p/MYH9 axis [19]; circCREBBP knockdown attenuates senescence via TGF-β2 [81]; SNHG5-enriched EVs reprogram autophagy and M2 polarization [111] | Genetic engineering of parent cells enables endogenous enrichment of specific therapeutic cargos during EV biogenesis [142]; miRNA-engineered EV immunotherapy targeting synovial inflammation [96]; circRNA-modified EVs modulate chondrocyte senescence and ECM anabolism [81,111] |
| **Small-Molecule / Drug-Engineered EVs** | Curcumin [78,105,141,156]; celecoxib [141]; anti-sense oligonucleotides against MMP-13 [78,105]; WNT3a [78,106] | Chondrocytes, joint tissues | Curcumin-loaded AD-MSC-sEVs suppress MMPs and boost aggrecan synthesis [78,105]; curcumin-primed BMSC-EVs reverse IL-1β-induced catabolic responses [156]; celecoxib-loaded EVs reduce synovial inflammation and oxidative stress [141]; EV-mediated WNT3a delivery overcomes cartilage matrix barriers to activate regenerative signalling [78,106]; anti-sense oligonucleotide-functionalized EVs achieve chondrocyte-targeted MMP-13 inhibition [78,105] | Small-molecule delivery via EV carriers overcomes bioavailability limitations [105,141]; EV-mediated WNT3a delivery penetrates dense cartilage ECM due to small EV size [78,106]; anti-sense oligonucleotide functionalization enables targeted MMP-13 silencing [78,105] |
| **Surface-Engineered / Charge-Modified EVs** | Charge-reversal modifications [132]; chondrocyte-targeting ligands [104,109]; chondrocyte-homing peptides [81] | Cartilage ECM, chondrocytes | Charge-reversal modifications enhance cartilage penetration [132]; chondrocyte-targeting EVs rejuvenate senescent chondrocytes by restoring metabolic function and reducing p16/p21 expression [104,109] | Surface engineering overcomes steric and electrostatic barriers within dense, negatively charged cartilage ECM [132]; active targeting enables delivery to deep-zone chondrocytes [104,109] |
| **Biomaterial-Integrated EV Delivery Systems** | EVs combined with hydrogels, microspheres, or scaffolds for sustained release [132,146]; TGF-β inhibitor-loaded EVs targeting subchondral bone [14]; macrophage-derived EV-functionalized systems for subchondral bone targeting [114]; gelatin methacryloyl (GelMA) hydrogels [151] | Chondrocytes, synoviocytes, subchondral bone (sustained local release) | Biomaterial-assisted delivery prolongs EV retention within the joint for sustained therapeutic effects [132,146]; engineered EVs delivering TGF-β inhibitor attenuate OA by targeting subchondral bone [14]; macrophage-derived EV-functionalized systems enable targeted delivery to subchondral bone [114] | Overcoming rapid EV clearance from synovial fluid via lymphatic drainage and phagocytic uptake [146]; spatial control of EV delivery across cartilage, synovium, and subchondral bone compartments [14,114]; biomaterial depots enable controlled, disease-responsive EV release [132,146] |
| **Organoid / Next-Generation EV Platforms** | 3D organoid-specific cargo distinct from 2D-cultured EVs [138]; cell-derived nanovesicles [158]; photosynthetic microorganism-derived EVs [160] | Joint tissue cells | Organoid-derived EVs recapitulate native tissue-level EV signalling with distinct therapeutic cargo [138]; bioreactor and 3D culture improve EV yield and functional consistency [158]; alternative biological sources offer innovative production avenues [160] | Next-generation EV platforms leveraging organoid technology for disease modelling and therapeutic EV production [138]; scalable manufacturing strategies addressing low native EV yield [158,160] |

**Abbreviations:** AD, adipose; ADAMTS, a disintegrin and metalloproteinase with thrombospondin motifs; ANXA1, Annexin A1; BM, bone marrow; Col II, collagen type II; COX2, cyclooxygenase-2; CSF-1, colony-stimulating factor 1; Cx43, connexin 43; ECM, extracellular matrix; ERK, extracellular signal-regulated kinase; ESCRT, endosomal sorting complex required for transport; EV, extracellular vesicle; FLS, fibroblast-like synoviocyte; FMRP, fragile X mental retardation protein; GelMA, gelatin methacryloyl; GPX4, glutathione peroxidase 4; GW4869, neutral sphingomyelinase inhibitor (blocks EV release); hEV, hybrid EV; HIF-1α, hypoxia-inducible factor 1α; IL, interleukin; IPFP, infrapatellar fat pad; LPS, lipopolysaccharide; m6A, N6-methyladenosine; MMP, matrix metalloproteinase; MSC, mesenchymal stem/stromal cell; mtDNA, mitochondrial DNA; NF-κB, nuclear factor kappa B; NLRP3, NLR family pyrin domain containing 3; NOX4, NADPH oxidase 4; OA, osteoarthritis; OB, osteoblast; OC, osteoclast; OTUD4, OTU deubiquitinase 4; PGE2, prostaglandin E2; PPARγ, peroxisome proliferator-activated receptor gamma; PRP, platelet-rich plasma; ROS, reactive oxygen species; SASP, senescence-associated secretory phenotype; sEV, small extracellular vesicle; SFRP5, secreted frizzled-related protein 5; SNHG5, small nucleolar RNA host gene 5; TGF-β, transforming growth factor beta; TIMP, tissue inhibitor of metalloproteinases; TNF-α, tumour necrosis factor alpha; TOMM20, translocase of outer mitochondrial membrane 20; TRAF6, TNF receptor-associated factor 6; UC, umbilical cord; VEGF, vascular endothelial growth factor; WJ, Wharton's jelly; YAP/TAZ, Yes-associated protein/transcriptional coactivator with PDZ-binding motif.

**Note:** Reference numbers in brackets correspond to the main manuscript reference list. EV populations are organized into four functional categories: Pathogenic EVs, Protective/Homeostatic EVs, MSC-Derived Therapeutic EVs, and Engineered/Modified EVs. EV function is inherently context-dependent: the same EV population may exert divergent effects depending on donor cell activation state, recipient cell microenvironment, OA disease stage, and EV subtype. See the main text for detailed discussion of each EV population and the underlying molecular mechanisms.
